# Deletion of a conserved transcript PG_RS02100 expressed during logarithmic growth in *Porphyromonas gingivalis* results in hyperpigmentation and increased tolerance to oxidative stress

**DOI:** 10.1371/journal.pone.0207295

**Published:** 2018-11-12

**Authors:** Priscilla L. Phillips, Leticia Reyes, Edith M. Sampson, Evan A. Murrell, Joan A. Whitlock, Ann Progulske-Fox

**Affiliations:** 1 Department of Microbiology and Immunology, A.T Still University of Health Sciences, Kirksville, Missouri, United States of America; 2 Department of Pathobiological Sciences, University of Wisconsin, Madison, Wisconsin, United States of America; 3 Department of Oral Biology, University of Florida, Gainesville, Florida, United States of America; East Carolina University Brody School of Medicine, UNITED STATES

## Abstract

The oral obligate anaerobe *Porphyromonas gingivalis* possesses a small conserved transcript PG_RS02100 of unknown function we previously identified using small RNA-seq analysis as expressed during logarithmic growth. In this study, we sought to determine if PG_RS02100 plays a role in *P*. *gingivalis* growth or stress response. We show that a PG_RS02100 deletion mutant’s (W83Δ514) ability to grow under anaerobic conditions was no different than wildtype (W83), but it was better able to survive hydrogen peroxide exposure when cultured under heme limiting growth conditions, and was more aerotolerant when plated on enriched whole blood agar and exposed to atmospheric oxygen. Together, these results indicate that PG_RS02100 plays a role in surviving oxidative stress in actively growing *P*. *gingivalis* and that *P*. *gingivalis’* response to exogenous hydrogen peroxide stress is linked to heme availability. Relative qRT-PCR expression analysis of *oxyR*, *trx-1*, *tpx*, *sodB*, *ahpC*, *dinF*, *cydB*, and *frd*, in W83Δ514 and W83 in response to 1 h exogenous dioxygen or hydrogen peroxide exposure, when cultured with varying heme availability, support our phenotypic evidence that W83Δ514 has a more highly primed defense system against exogenous peroxide, dioxygen, and heme generated ROS. Interestingly, W83Δ514 turned black faster than W83 when cultured on whole blood agar, suggesting it was able to accumulate heme more rapidly. The mechanism of increased heme acquisition observed in W83Δ514 is not yet known. However, it is clear that PG_RS02100 is involved in modulating the *P*. *gingivalis* cell surface in a manner related to survival, particularly against oxidative stress.

## Introduction

*Porphyromonas gingivalis* is a non-motile asaccharolytic Gram-negative obligate anaerobe commonly found in the oral cavity. It is recognized as a major pathogen of severe adult periodontitis [1] and implicated in systemic inflammatory conditions including cardiovascular disease [2–4] and poor pregnancy outcomes [5, 6]. As a pathobiont, with dual roles as a commensal organism and a pathogen, *P*. *gingivalis* has evolved multiple mechanisms to persist in its host and disseminate [7]. Those factors that facilitate its colonization and growth in the oral cavity, vascular endothelium, or deeper tissues are presumably host site dependent. For example, as an obligate anaerobe dependent on the availability of heme (iron and protoporphyrin IX), *P*. *gingivalis* is better adapted to the environmental conditions of the subgingival space, particularly as a constituent of oral biofilms. However, *P*. *gingivalis* is commonly found in the ever-fluctuating conditions of all sites of the oral cavity, and thus must be able to tolerate oxygen in order to persist. Furthermore, heme limitation/starvation, typical in health, transitions to heme excess during active periodontal disease or other injuries to the host epithelium. To better survive this fluctuation of heme feast or famine, *P*. *gingivalis* acquires and stores heme on its cell surface. The acquisition of iron mediated through surface storage of heme may become problematic when iron, typically in the ferrous state under anaerobic conditions, is exposed to oxygen. For example, free iron donates electrons to oxygen, forming reactive oxygen species (ROS). *P*. *gingivalis* is also able to invade host cells and disseminate, primarily though the vascular system, to deeper tissues [6]. In this scenario, *P*. *gingivalis* must also be able to resist intracellular and extracellular ROS produced by the host. Identifying factors that facilitate survival in the dynamic environment of the host will increase our understanding of how this obligate anaerobic pathogen persists in various human tissues.

PG_RS02100 is a gene of unknown function, conserved among *P*. *gingivalis* strains, that we previously identified as a small transcript expressed only during logarithmic growth, irrespective of heme availability [8]. In our previous study we sought to identify small transcripts that may potentially function as regulatory genes that facilitate rapid response to environmental changes during active growth. In this study, we sought to determine if PG_RS02100 plays a role in facilitating survival under conditions of oxidative stress. Here, we report that a PG_RS02100 deletion mutant created in *P*. *gingivalis* strain W83 (W83Δ514) displayed a pleiotropic phenotype including hyperpigmentation, indicative of accelerated heme acquisition and storage, and increased tolerance to oxidative stress.

## Materials and methods

### Construction of mutant and complement strains

PCR mediated gene replacement was used to create a deletion mutant of the putative PG_RS02100 gene referred to in this study as W83Δ514. Custom designed primers (S1 Table) and *P*. *gingivalis* strain W83 genomic DNA were used to generate PCR fragments containing sequences flanking the target site for deletion mutation. A PCR fragment containing the Erm cassette (*ermF/ermAM*) was generated using plasmid DNA encoding the Erm cassette (mutant selectable marker) as the template. The three PCR generated fragments were fused by sequential denaturation, hybridization and PCR extension/amplification. The full-length fusion product was gel purified and electroporated into *P*. *gingivalis* strain W83. Electroporated cells were plated onto modified blood agar plates (BAP) with 10 μg/ml erythromycin and incubated for up to 10 days. Positive transformants that underwent homologous recombination were selected and screened by PCR analysis and verified by DNA sequencing of isolated genomic DNA.

In addition, custom designed primers (S1 Table) and W83Δ514 genomic DNA were used to generate PCR products containing sequences flanking the target site in order to complement the deletion mutation in W83Δ514 and restore expression of PG_RS02100 in the mutant by directed insertion mutation. A PCR fragment containing the Bacteroides *TetQ* gene was generated using plasmid DNA encoding TetQ (complement selectable marker) as the template. The three PCR fragments were fused by sequential denaturation, hybridization and PCR extension/amplification. The full-length fusion product was gel purified and electroporated into the W83Δ514 deletion mutant. Electroporated cells were plated onto modified BAP with 1 μg/ml tetracycline and incubated for up to 10 days. Positive transformants that underwent homologous recombination were selected and screened by PCR analysis. The complemented strain was both erythromycin (ErmF) and tetracycline (TetQ) resistant.

DNA sequencing was also used to assess the sequence of up-stream and downstream genes (crossover regions) within the mutant and complement strains to verify that no unintended mutation was introduced during recombination.

### Bacterial culture and cultural characteristics

Freezer stocks of *P*. *gingivalis* strains, wildtype and mutants, were plated onto modified blood agar plates (BAP: Tryptic soy agar supplemented with 5 μg/ml yeast extract, 0.5 μg/ml L-cysteine hydrochloride, 1 μg/ml Vitamin K1, 5 μg/ml hemin chloride from bovine, 5% whole sheep blood Alsevers), and appropriate antibiotics, and cultured for 4 days under anaerobic conditions (10% H_2_, 5% CO_2_, 85% N_2_) at 37˚C. *P*. *gingivalis* was streaked for isolation and isolated colonies used to prepare broth cultures. Plate cultures were passed no more than three times before fresh plates were prepared from freezer stocks. *P*. *gingivalis* was cultured overnight in pre-reduced heme rich (HR; 5 μg/ml hemin) modified TSB media (TBS supplemented with 5 μg/ml yeast extract, 0.5 μg/ml L-cysteine hydrochloride, 1 μg/ml Vitamin K1) or heme limiting (HL; 0.001 μg/ml hemin) modified TSB media, with or without antibiotics as appropriate, under anaerobic conditions at 37°C. For heme starvation (HS) conditions, overnight HL-cultures (stationary phase) were pelleted and suspended in fresh modified TSB (without hemin) daily for 2 days before cells were collected. Because we previously determined that PG_RS02100 was expressed in W83 during logarithmic growth using microarray and RNA-seq analysis [8], overnight cultures were used to inoculate 50 ml pre-reduced media with an initial optical density (OD_550nm_) between 0.1–0.2, and cultured to log-phase for all assays unless otherwise noted. Cultural characteristics and Gram stains were assessed for all cultures prepared in this study.

Growth curves were generated for both wildtype and mutant strains, under both HR and HL conditions, over the course of 26 h. When cultures reach OD_550nm_ >1, culture samples are diluted 10-fold before determining its optical density, and mathematically converted before plotting. These cultures were prepared by inoculating appropriate media to an initial OD_550nm_ between 0.1–0.15 using overnight starter cultures.

All cultures used for RNA and molecular analyses were incubated until they reached late log-phase at an OD_550nm_ of (1–1.5) except for the HS condition, which were prepared as described above. For RNA analysis, cells were immediately harvested or were treated for 1 h under conditions described below and in Table 1. During the treatment period, all cultures were kept under anaerobic conditions at 37°C except the O_2_ with shaking treatment where the flasks were placed in a shaker (225 rpm) under aerobic conditions at 37°C for 1 h. Bacterial cell pellets collected from 20 ml of culture were each suspended in 3 ml RLT buffer (Qiagen; Gaithersburg, MD, USA) and the RNA isolated for subsequent qRT-PCR analyses.

**Table 1:** Fold-change in expression (transcription level) of gene targets in W83Δ514 mutant relative to expression in wildtype strain W83.

Gene ID	Gene locus / Old tag	HR-HR 7.7 μM hemin chloride	HL-HL 2 nM hemin chloride	HL-HS 2-day hemin starvation	HR-HR 1 h 0.25 mM H_2_O_2_	HL-HL 1h 0.25 mM H_2_O_2_	HR-HR 1 h Atmospheric O_2_ with shaking
OxyR	PG_RS01210 / PG0270	-5.1337	1.681792	4.40762	1.965641	1.641483	2.522754
Trx	PG_RS00170 / PG0034	-1.197478	-1.464085	12.295	4.213444	2.505328	1.500038
Dps	PG_RS00405 / PG0090	1.180992	3.363585	-1.796264	-1.156688	1.853176	-4.941674
Tpx	PG_RS07610 / PG1729	-1.319507	7.963117	1.189207	2.44528	1.699369	-1.389918
CydB	PG_RS03960 / PG0899	-3.470154	1.547564	1.140763	3.837056	2.297396	2.566851
SodB	PG_RS06820 / PG1545	-1.113421	2.29739	1.296839	3.127479	3.363585	3.317278
AhpC	PG_RS02725 / PG0618	-1.156688	-1.031683	6.956363	6.588728	7.963117	4.59479
DinF	PG_RS07215 / PG1640	-2.13613	-2.353813	-1.905275	2.042024	2.034959	4.691339
FrdB	PG_RS07115 / PG1614	-1.630144	1.641483	2.566851	-1.31494	-1.464085	1.342572

**Table 1** values are reported as the average fold-change in expression between two independent experiments. A value less than 1 indicates decreased expression in the mutant relative to wildtype.

### Gingipain assay

The gingipain assay used in this study, described in more detail in S1 Appendix, was modified from the protocol described by Potempa and Nguyen [9]. Briefly, gingipain substrate was prepared to assess for Kgp activity (L-Lysine-p-nitroanilide dihydrobromide; Sigma L7002) and Rgp activity (BAPNA; Sigma B4875). *P*. *gingivalis* was cultured in HR-media for 3 days anaerobically at 37°C for maximal gingipain production, as it has been previously reported that gingipain protein maturation is maximal at stationary phase [10]. Cell pellets from 2 ml fractions of each culture were collected and the spent media (filtered cell-free supernatants) reserved. Cell suspended in buffer were diluted to 100%, 50%, 25%, 12.5%, 6.25%, 3.125% and spent media were diluted to 100%, 50%, 25%, 12.5%, 0% (negative control). Each well of a 96-well plate loaded with 90 μl assay buffer and 100 μl of sample (two replicate wells) was incubated at 37°C for 10 min ensure reduction of cysteine residues, and 10 μl of appropriate substrate solution was added to each well (except blanks) to produce a final concentration of 0.5 mM substrate. The absorbance at 410 nm was recorded using a temperature-regulated kinetic spectrophotometric microplate reader and collected continuously over 2 to 30 min. To determine the initial rate of hydrolysis, the linear portion of the absorbance versus-time curve was calculated for each sample and adjusted by the rate of non-enzymatic substrate hydrolysis observed in the negative control wells. Since *P*. *gingivalis* does not significantly produce other Arg-specific or Lys-specific proteases [11], the rate of L-BAPNA and Ac-Lys-pNA hydrolysis is considered a direct measurement of gingipain activity. Moreover, the rate of L-BAPNA hydrolysis represents the sum of contributing forms of Rgp proteases expressed in *P*. *gingivalis*.

### Atmospheric O_2_ stress plate assay

Six independent experiments were performed. During each independent experimental trial, two log-phase HR broth cultures were prepared of each strain (OD_550nm_ = 0.7–1) and placed on ice to stop growth. Ten-fold serial dilutions were prepared (using 900 μl 1x PBS in microfuge tubes). 0.1 ml of appropriate dilutions were spread on modified BAP (two plates per dilution tube) and incubated at room temperature (RT) under aerobic conditions. Plates were prepared in batches and initial (t_o_) time recorded to maintain consistent assay timing. Plate sets at each assayed time-point were transferred to anaerobic conditions at 37°C. Surviving colony-forming units (CFU) were counted and recorded.

### H_2_O_2_ stress growth curve assay

Four independent experiments were performed. During each independent experimental trial, overnight broth cultures (two cultures per strain per condition) were used to prepare starter cultures with an initial OD_550nm_ of 0.15. All cultures were maintained under anaerobic conditions at 37°C throughout. Because this study showed that each strain (wildtype vs mutant) had a similar growth rate under each media condition, after 7 h of incubation, freshly prepared 50 mM H_2_O_2_ (Sigma 31642) prepared in media was added to all cultures to the desired final concentration (0.5 mM, 0.25 mM, 0.125 mM, 0 mM). 1 ml samples were collected from each culture at each assay time-point, before and after addition of H_2_O_2,_ and the OD_550nm_ recorded. CFUs were also determined for all initial (t_0_) cultures and before addition of H_2_O_2_ (t_7h_).

### Extracellular H_2_O_2_ generation assay

Bacterial cell pellets (1.5–2 x 10^7^ cells) collected from log-phase cultures (OD_550nm_ = 1.0–1.3) were suspended in reaction buffer and freeze thawed twice (20 min -80°C / 20 min RT). The concentration of cellular H_2_O_2_ was then assayed using an Amplex red assay kit (Molecular Probes, Eugene, OR, USA) according to the manufacturer’s protocol. CFUs were determined for all cultures to normalize data.

### RNA isolation

Total RNA was isolated (including transcripts <200 nt) for northern analysis using the mirVana miRNA isolation kit (Ambion, Foster City, CA, USA) and treated with Turbo DNase (Invitrogen, Life Technologies, Carlsbad, CA, USA) as per manufacturers instructions. In contrast, total RNA for qRT-PCR analysis was prepared using a combination of TRIzol lysis and RNeasy (Qiagen) extraction [12]. Briefly, 750 μl of TRIzol LS was added per 250 μl RLT-bacterial suspension and incubated for 5 min. 200 μl of cold chloroform was added to each 1ml cell lysate, mixed, incubated for 2 min, centrifuged 12,000 x g for 15 min at RT. A 450 μl of the aqueous phase was collected and an equal volume of 70% ethanol was added per sample. Samples were then applied to RNeasy Mini spin columns (Qiagen) and the RNA was isolated as per manufactures instruction and treated with Turbo DNase (Invitrogen).

### PG_RS02100 RNA expression analysis

Total RNA extracted from HL and HR log-phase cultures of W83Δ514 and W83 were used as the template for qRT-PCR analysis to 1) verify loss of expression in the mutant 2) verify expression of PG_RS02100 during log phase, and 3) compare expression of PG_RS02100 during HL and HR growth conditions (S3 Table). To normalize the data and determine the relative expression level of the PG_RS02100 transcript, primers to bacterial 16S rRNA was used to generate a standard curve using known concentrations of genomic *P*. *gingivalis* DNA (S2 Table).

### qRT-PCR expression analysis

Single strand cDNA was generated from isolated total RNA samples using iScript cDNA synthesis kit (Bio-Rad) as per manufacturer instructions. The qRT-PCR reactions were prepared using IQ SYBR Green qRT-PCR kit (Bio-Rad) as per manufacturers instructions with custom designed primer pairs to select gene targets (S2 Table) and run on iQ5-icycler (Bio-Rad). Each primer set was designed to generate 200 bp PCR amplicons except those to PG_RS02100, which produce a 50 bp PCR amplicon and was used to qualitatively verify wildtype expression and loss of expression in the mutant. The qRT-PCR data was analyzed by the standard comparative Ct method [13]. To determine the fold-change in expression value for each target gene, the 2^-ΔΔCt^ was calculated. If the fold-change value was less than 1, indicating reduction in expression in the mutant relative to the wildtype, the negative inverse of the fold-change value was calculated. A change in relative expression of 3-fold or greater was selected as the threshold in our analysis as a notable difference in expression of a target gene in the mutant W83Δ514 relative to wildtype W83. Changes below 2-fold were considered similar levels of expression.

### Northern analysis

Biotinylated probe to PG_RS02100 (5’-TGATGAATACCGAACAGGCTGAAAAATGCATCGCTACACCTGCCGGCAGA-3’) was prepared from custom designed QuickLC purified 50 nt single stranded DNA oligonucleotides (Thermo-Fisher Scientific). The oligonucleotides were biotinylated using a BrightStar Psoralen-Biotin nonisotopic labeling kit (Ambion). Total *P*. *gingivalis* RNA was isolated from cultures grown to log or stationary phase in HR or HL media. Purified and DNase treated RNA was heat denatured for 15 min at 65°C and size fractionated in 10% TBE-urea acrylamide gels (Invitrogen) for 2 h at 100 V. Each lane was loaded with 5 μg of purified RNA per lane. The molecular weight standard Century™ Plus BrightStar Biotinylated RNA marker (Ambion AM7180) was loaded onto the gel. Size fractionated RNA was transferred by electro-blotting (Transblot SD; Bio-Rad) at 10 V for 1 h onto BrightStar-Plus nylon membranes (Ambion). Psoralen-biotin probe hybridization onto the blots was carried out in ULTRAhyb ultrasensitive solution (Ambion) overnight at 42°C. Blots were washed using NorthMax Low Stringency Buffer (Ambion) at 42°C for 2 min. Northern blots were developed using streptavidin-alkaline phosphatase conjugate and detected using CDP-Star chemiluminescent substrate according to manufacturer’s protocols (BrightStar BioDetect, Ambion). The blots were imaged every 20 min using ChemiDoc imaging system (Bio-Rad).

### *In silico* analyses

For this study, we originally selected a transcript that we had previously predicted to encode a regulatory sRNA [8], for mutational analysis based on the following criteria: 1) it was positively identified by both microarray and Illumina sequencing methods; 2) it was located in the intergenic region (IGR) between two genes whose stop codons were positioned in proximity to the IGR to minimize the possibility of polar effects of neighbor gene expression in the mutant constructs (S4 Table); and 3) bioinformatic analysis using transcription terminator prediction program TransTermHP identified a putative Rho-independent terminator (S4 Table). BLAST and pfam taxonomy analysis [14] was performed.

## Results

### Comparison of the relative expression of PG_RS02100 in W83 under heme rich and heme limiting growth conditions

**Fig 1:**
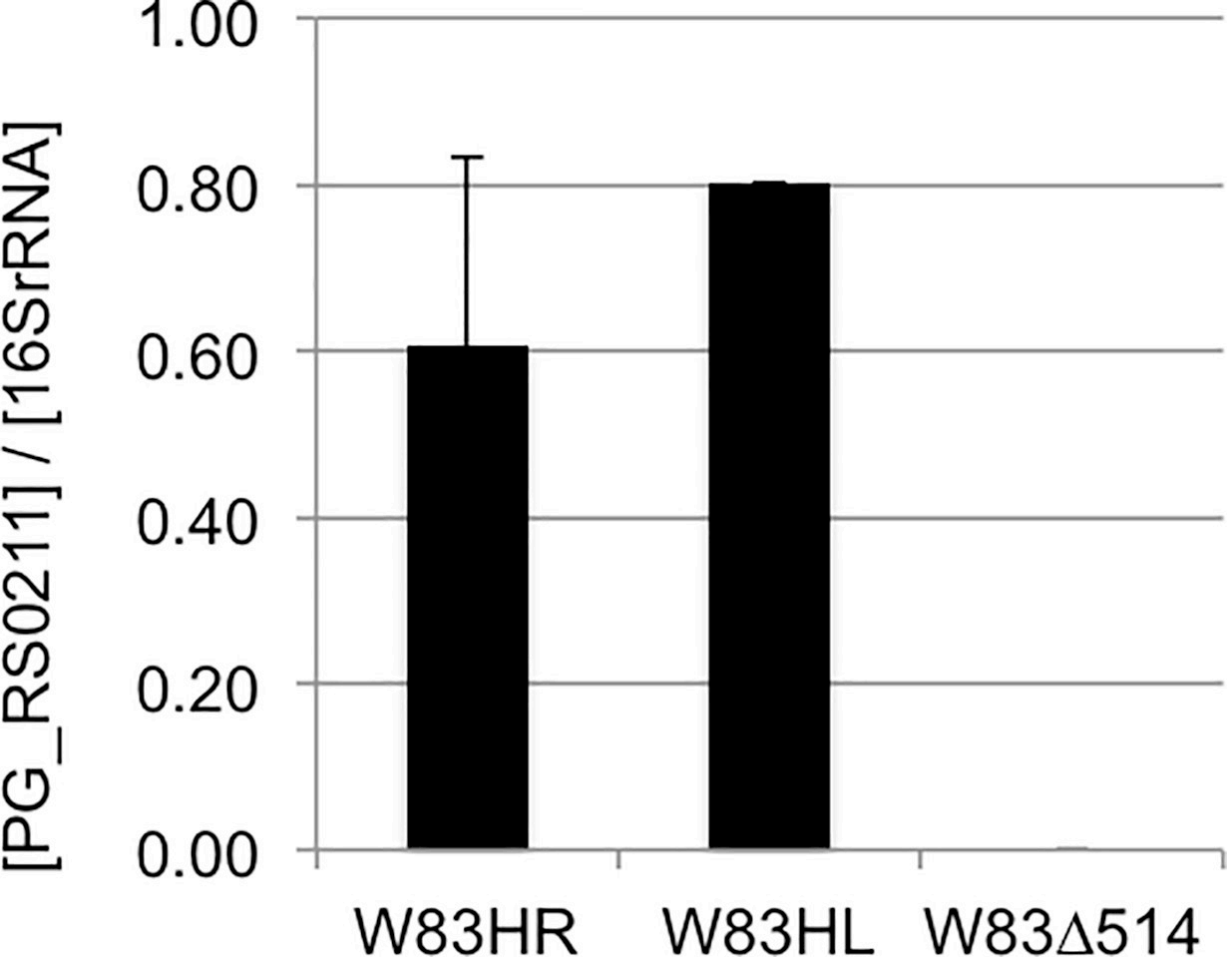
PG_RS02100 RNA expression. The data is reported as a ratio of the relative concentration of PG_RS02100 transcript divided by the relative concentration of 16S transcript within each total RNA sample. Student t-test suggests there was no statistical difference (p-value = 0.347) in the relative concentration of PG_RS02100 in W83 between HL or HR growth conditions at log-phase. Expression of PG_RS02100 was not detected in W83Δ514.

Quantitative RT-PCR expression analysis using total RNA isolated from W83Δ514 and W83 confirmed that this transcript was expressed in W83 during logarithmic phase at similar levels under HL and HR conditions, and is not expressed in W83Δ514 (Fig 1). This data supports our Illumina sequencing findings which showed that PG_RS02100 was expressed during log-phase, irrespective of heme availability [8].

### *In silico* and northern analyses of PG_RS02100

**Fig 2:**
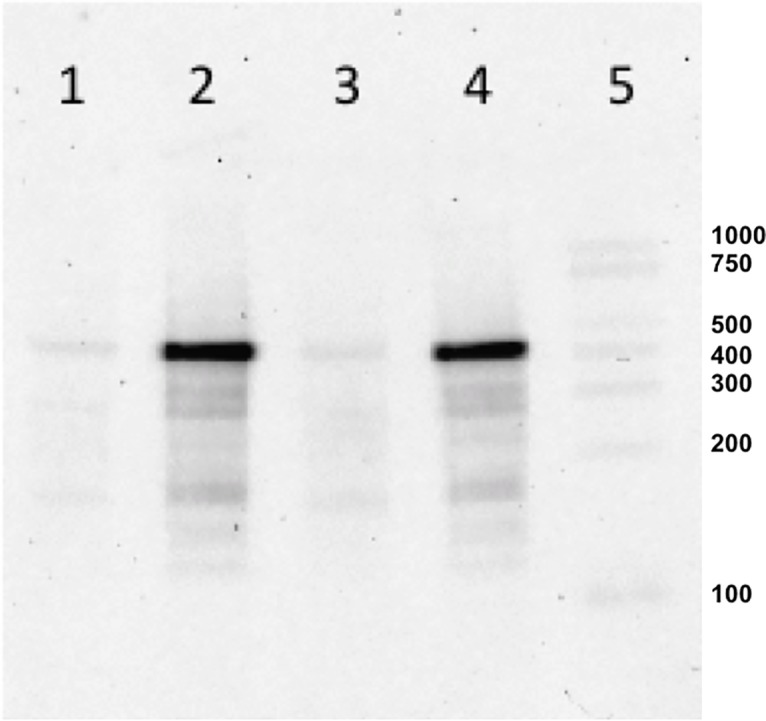
Northern blot analysis. 10% TBE-urea polyacylamide gel lanes were loaded with purified W83 RNA isolated from the following culture conditions 1) HL-Stationary phase 2) HL-Log-phase 3) HR-Stationary-phase 4) HR-Log-phase 5) RNA Standard.

*In silico* analysis predicted a 429 nt transcript, from the 5’end identified by both illumina sequencing and microarray, to the *in silico* predicted Rho-independent terminator. Northern analysis verified the RNA transcript size and confirmed that a single transcript was expressed during logarithmic but not stationary phase culture (Fig 2). The initial predictive *in silico* analysis of this transcript was supported using the Oralgene genome database and small noncoding RNA analysis tool (http://www.oralgen.org/). A submitted query using this tool reported a matching intergenic sequence (ISG), number 164, identified as conserved (nucleotide sequence) within *P*. *gingivalis* species but not found in other microbial species in this database. On the positive strand, no promoters and one terminator were identified. On the negative strand, no promoter or terminator was identified. Subsequent updates to the NCBI database identified a 403 nt putative open reading frame designated PG_RS02100 at this locus in the *P*. *gingivalis* strain W83 genome (NC_002950.2) between PG_RS02095 and PG_RS02105, presuming *P*. *gingivalis* identifies the UUG codon, which is reported to be rarely used in *E*. *coli* [15]. The putative translated amino acid sequence (WP_012458318.1) contains a conserved putative domain of unknown function (DUF4248/PF14053) reported to be typically found in small putative proteins between 73–86 amino acids in length. BLAST and pfam taxonomy analysis (Query IDs WP_012458318.1 and DUF4248 respectively) identified amino acid sequence matches to 55 species (205 organisms) in the phylum CFB, with 52 in the order Bacteriodales [Families: 11 Porphyromonadaceae, 29 Bacteriodaceae, 11 Paraprevotella] (S1 Fig). Amino acid identity was restricted to the putative DUF4248 domain in organisms outside the Porphyromonas genus. With regard to the flanking genes, PG_RS02095 found upstream is predicted to encode a tRNA epoxyqueuosine reductase QueG containing a 4Fe-4S double cluster binding domain, and PG_RS02105, found downstream is predicted to encode an amino acid lyase containing a pyridoxal 5’-phosphate binding pocket. This neighboring gene arrangement is conserved across all *P*. *gingivalis* strains with complete annotated genomes in the NCBI database. The sequence spanning the IGR between between PG_RS02095 and PG_RS02105 in the mutant (W83Δ514) and complemented strains (W83Δ514-Complement) can be found in S5 Table.

### Comparison of culture characteristics between mutant and wildtype strains

**Fig 3:**
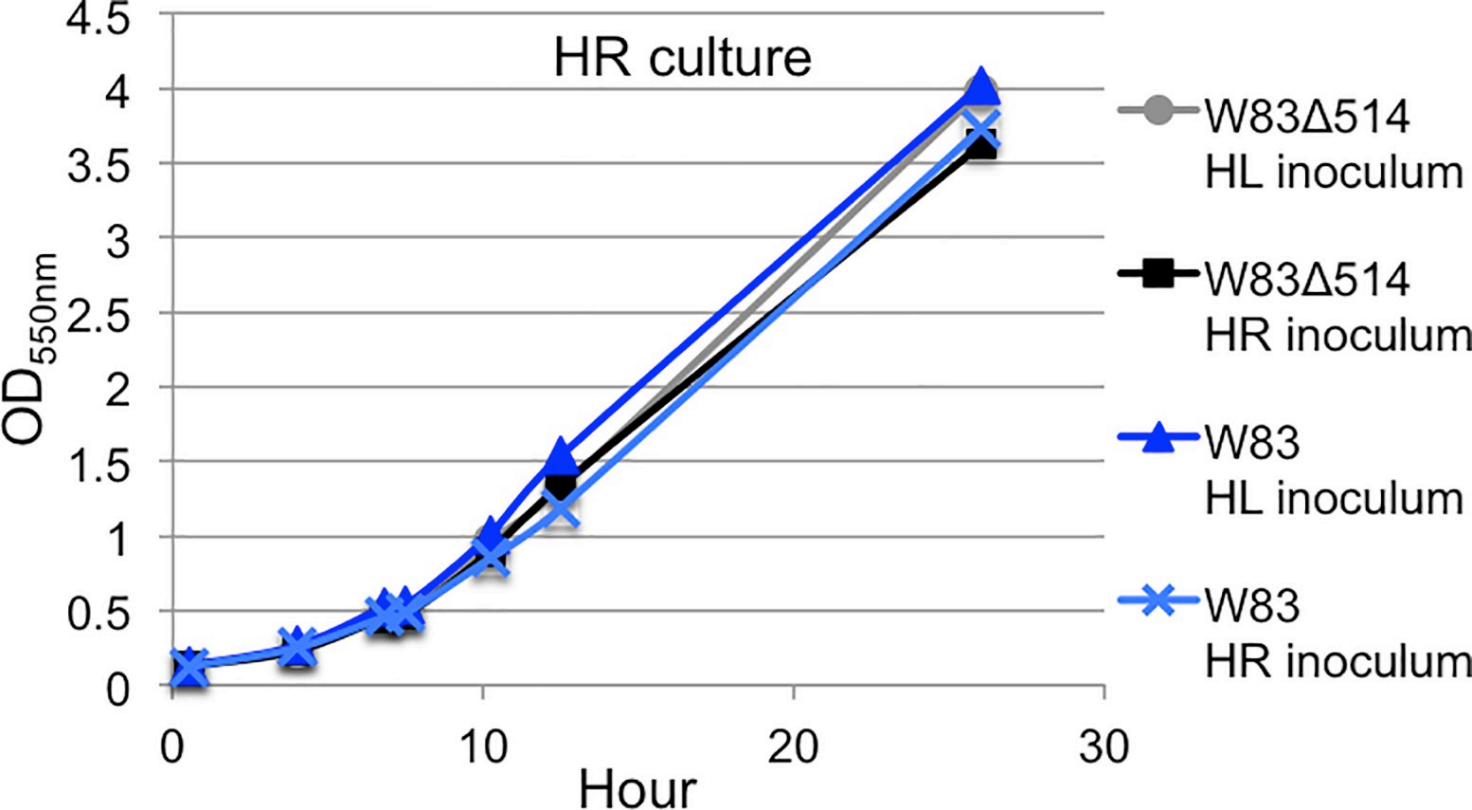
Growth curve of *P*. *gingivalis* strain W83 and W83Δ514 under heme rich conditions. Wildtype (blue) and PG_RS02100 deletion mutant (black) strains were cultured in pre-reduced HR-media inoculated (starting OD_550nm_ = 0.1) with stationary bacteria that were cultured in HR or HL media. The optical density was recorded at the specified time points over 26 h and reported as the average optical density (n = 2 per curve). The results shown in this representative figure were confirmed in three separate experiments.

**Fig 4:**
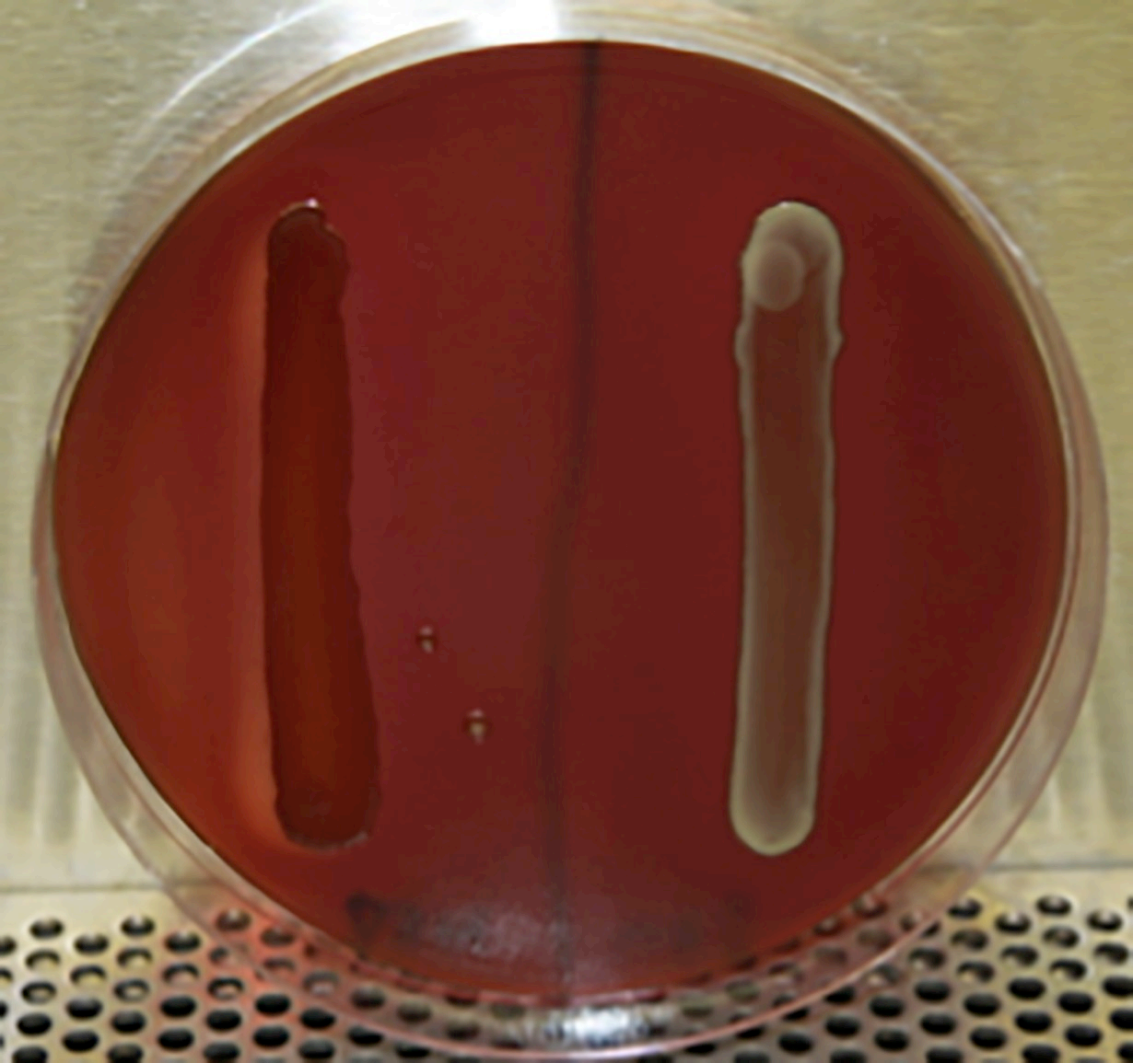
W83Δ514 hyperpigmentation on whole blood BAP. Overnight broth cultures streaked on modified BAP containing whole (non-lysed) blood after 3-day incubation (Left W83Δ514, Right W83).

Wildtype *P*. *gingivalis* strain W83 and PG_RS02100 deletion mutant W83Δ514 were cultured to determine if there were any differences in their rate of growth. No notable difference was observed in the growth rate between the mutant and wildtype strains under HR-culture conditions (Fig 3). However, there was a notable decrease in growth rate under continuous HL-culture conditions compared to growth under continuous HR-culture conditions. Interestingly, an increased rate of acquiring pigmentation (hyperpigmentation) was observed for W83Δ514 colonies cultured on modified blood agar plates containing whole sheep’s blood compared to wildtype (Fig 4). This obvious difference in pigmentation acquisition was not observed when these strains were plated onto modified blood agar plates containing laked blood (i.e., hemolyzed red blood cells). Under this condition, all strains rapidly acquired pigmentation.

### Comparison of gingipain activity between mutant and wildtype strains

**Fig 5:**
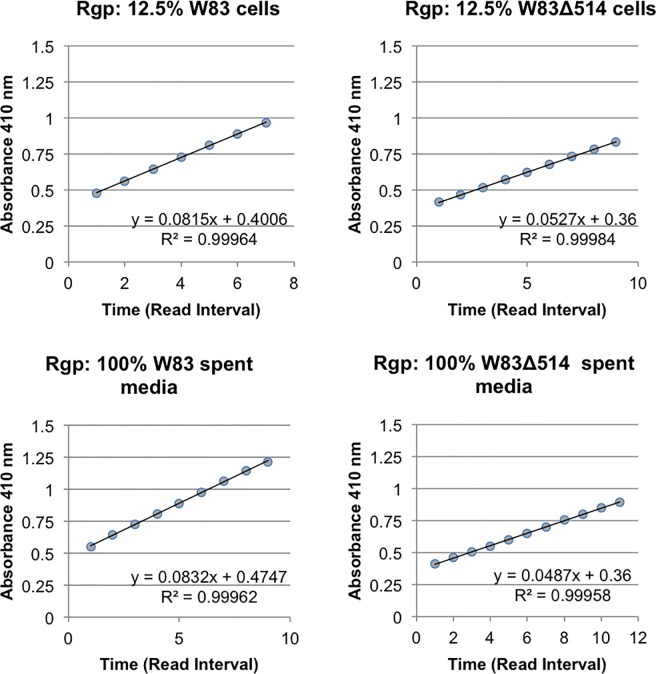
Whole cell and spent media from stationary cultures were assayed for Rgp substrate hydrolysis activity using the gingipain assay. Absorbance at 410 nm was collected at regular intervals over a 30 min period and the interval within the linear range was plotted as the average absorbance.

**Fig 6:**
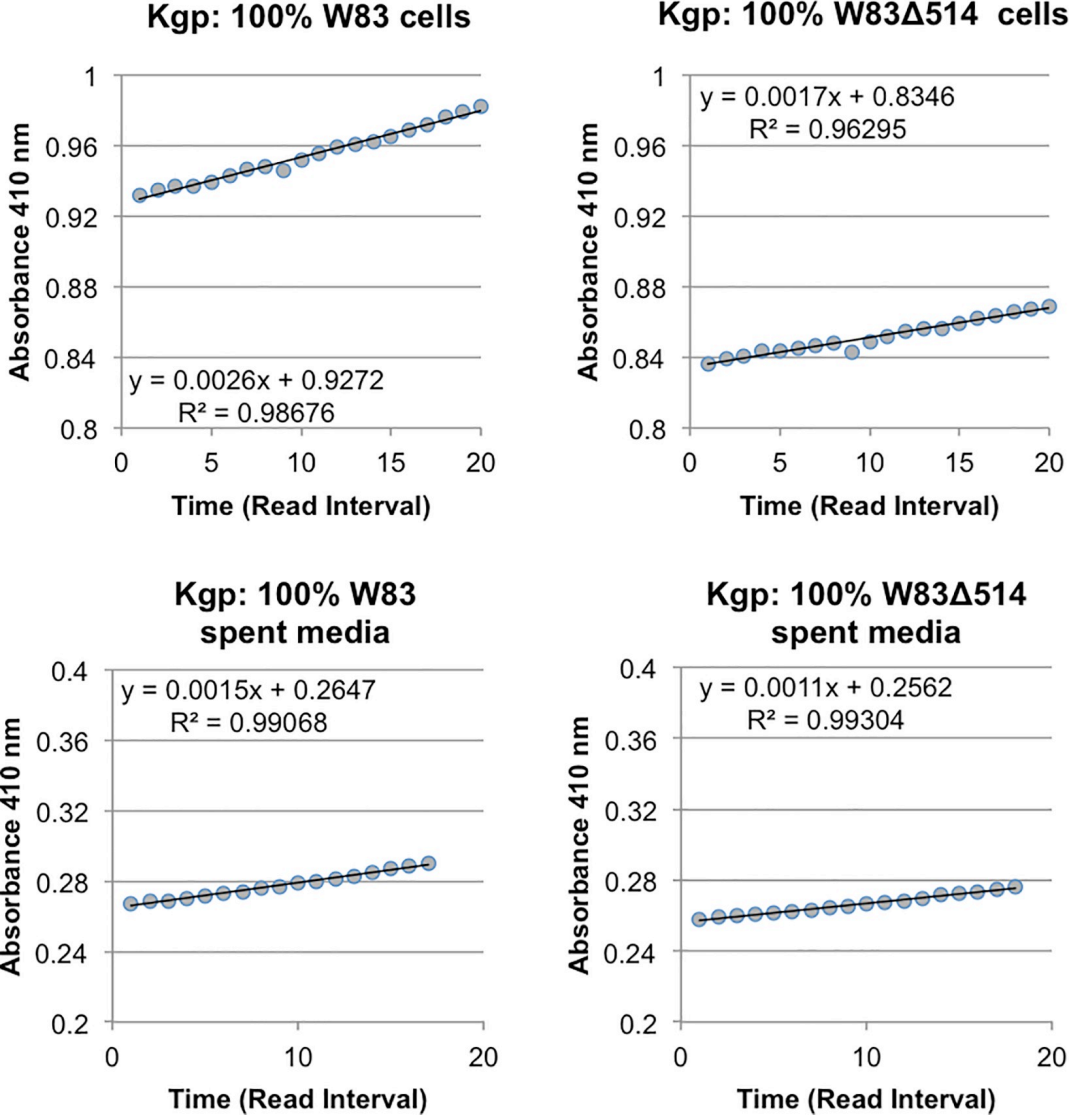
Whole cell and spent media from stationary cultures were assayed for Kgp substrate hydrolysis activity using the gingipain assay. Absorbance at 410 nm was collected at regular intervals over a 30 min period and the interval within the linear range was plotted as the average absorbance.

Stationary cultures in HR were collected and both the cells and cell-free supernatants were assessed for the rate of Rgp (Fig 5) and Kgp (Fig 6) substrate hydrolysis. Despite a slightly higher spectrophotometric optical density of W83Δ514 compared to W83 (up to 110%) during the performance of this assay, the relative rate of substrate hydrolysis was consistently lower in the mutant for both substrates with each corresponding dilution. The difference in the rate of hydrolysis in the mutant samples ranged between 65–85% of the rate found in the corresponding wildtype sample. The decreased hydrolysis rate was more pronounced in the sample dilutions that did not deplete the substrate within 20 min, compared to more concentrated samples that detectably hydrolyzed all the substrate in approximately 5 min (absorbance saturation). Regression analysis was performed [16] and the equation of the line are shown on each graph (Fig 5, Fig 6). The regression coefficient for wildtype (~0.08) is almost twice that of mutant (~0.05) indicating the absorbance (Rgp activity) increases at a faster rate in the wildtype for both the cells and spent media. The coefficient of determination (R^2^) shows the highly predictive value of the equation (>99.9% confidence) for each graph (Fig 5), indicating the observed differences in the regression coefficient are statistical significant. The regression coefficient for wildtype (0.0026 cells; 0.0015 spent media) is larger than that of mutant (0.0017 cells; 0.0011 spent media) indicating the absorbance (Kgp activity) increases at a slightly faster rate in the wildtype. The coefficient of determination (R^2^) shows the highly predictive value of the equation (96.3% - 99.3% confidence) for each graph (Fig 6), but considering the very small difference in rate and the room for error as indicated by the coefficient of determination, we are not confident that there is statistical significance between the observed differences in the regression coefficient for Kgp. However, the greater cellular density in the mutant cultures gives greater confidence that this slightly higher level of Kgp activity observed in the wildtype is real. These data indicate that under heme rich conditions, stationary cultures of the mutant had decreased gingipain activity relative to the wildtype.

### Comparison of oxidative stress tolerance between mutant and wildtype strains

**Fig 7:**
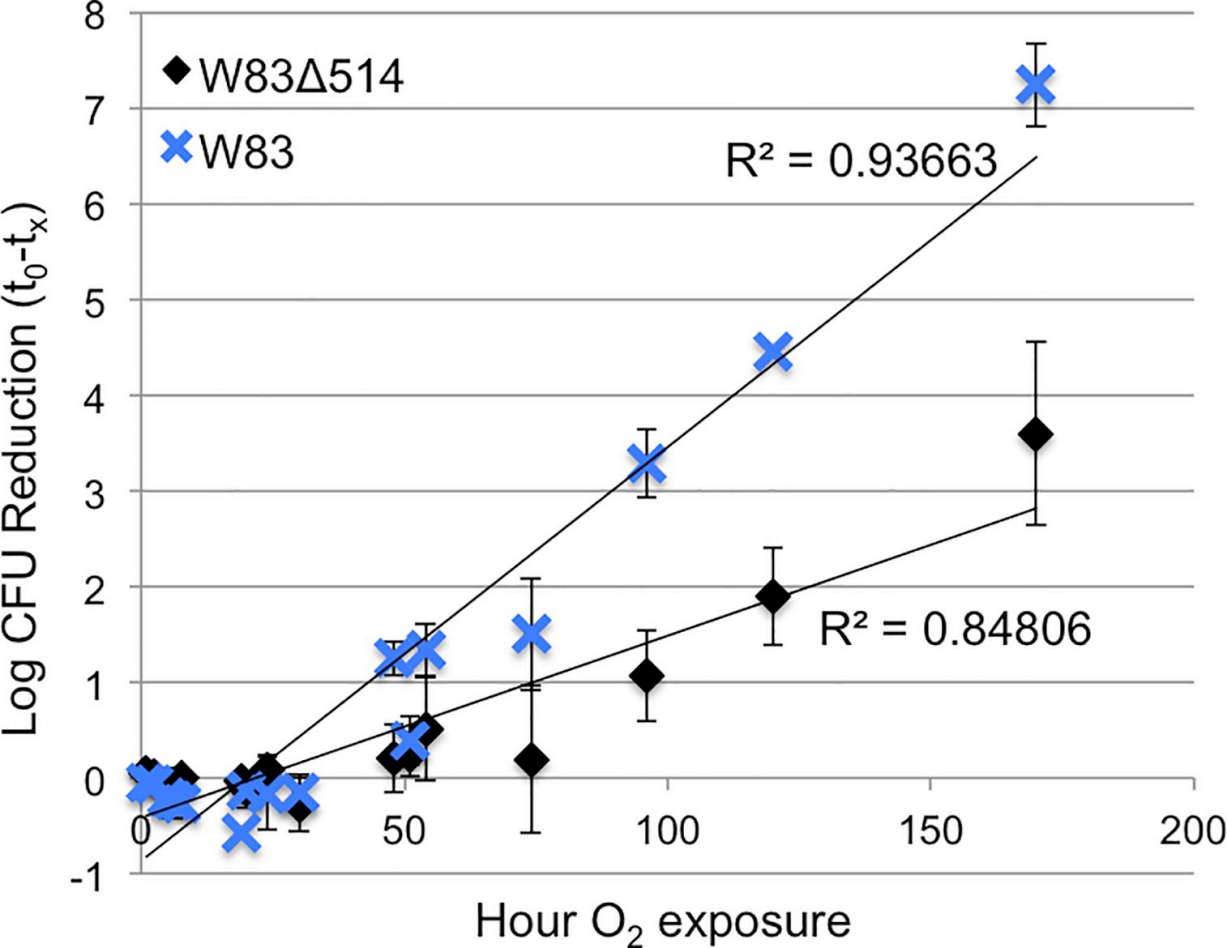
W83Δ514 is more resistant to atmospheric oxygen stress than wildtype W83. Serial dilutions of log-phase HR-cultures were plated onto enriched blood agar (5μg/ml hemin supplemented, 1μg/ml Vitamin K_1_) and exposed to atmospheric oxygen at RT before transfer to standard anaerobic conditions. The number of cells that survived was collected from five independent experiments, with two biological replicates for each strain within each experiment, and the number that died due to continuous dioxygen exposure relative to the initial CFU plated was plotted as the average Log CFU reduction ± SD. By 170 h of exposure, the CFUs for W83 were reduced by 7–8 logs while W83Δ514 CFUs were reduced 3–4 logs.

**Fig 8:**
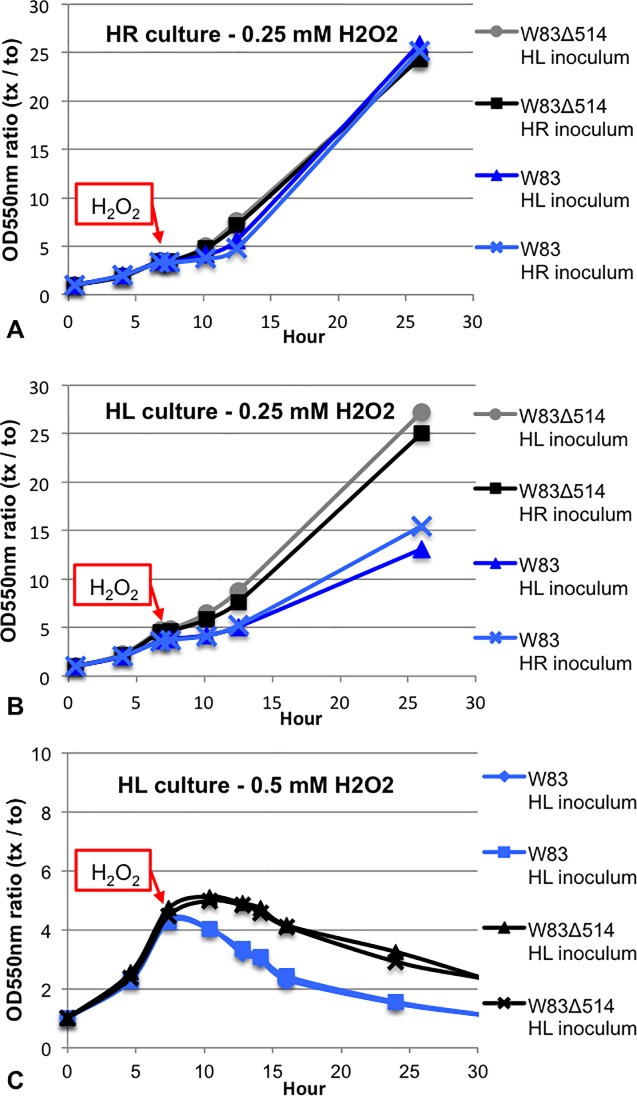
W83Δ514 mutant is more resistant to hydrogen peroxide stress than wildtype strain W83 when continuously cultured under heme limitation. Data is reported as a ratio of average OD (t_x_) to average starting OD (t_0_) in each survival curve (n = 2 per curve). A) Pre-reduced HR-media were inoculated (starting OD_550nm_ = 0.12–0.13) with bacteria from HL or HR stationary cultures. H_2_O_2_ was added when the 25 ml cultures reached early log-phase (OD_550nm_ = 0.5–0.7) to a final concentration of 0.25 mM and the optical density was recorded at the specified time points over 24 h. B) Pre-reduced HL-media was inoculated and exposed to 0.25 mM H_2_O_2_ in the same manner. The results with exposure to 0.25 mM H_2_O_2_ shown in this representative figure were confirmed in four separate experiments for a total of four growth curves per strain and condition. C) Pre-reduced HL-media was inoculated (starting OD_550nm_ = 0.14–0.17) with bacteria from HL-stationary-cultures and exposed to 0.5 mM H_2_O_2_ in the same manner for 30.2 h. The results with exposure to 0.5 mM H_2_O_2_ shown in this representative figure were confirmed in two separate experiments for a total of four growth curves per strain and condition.

**Fig 9:**
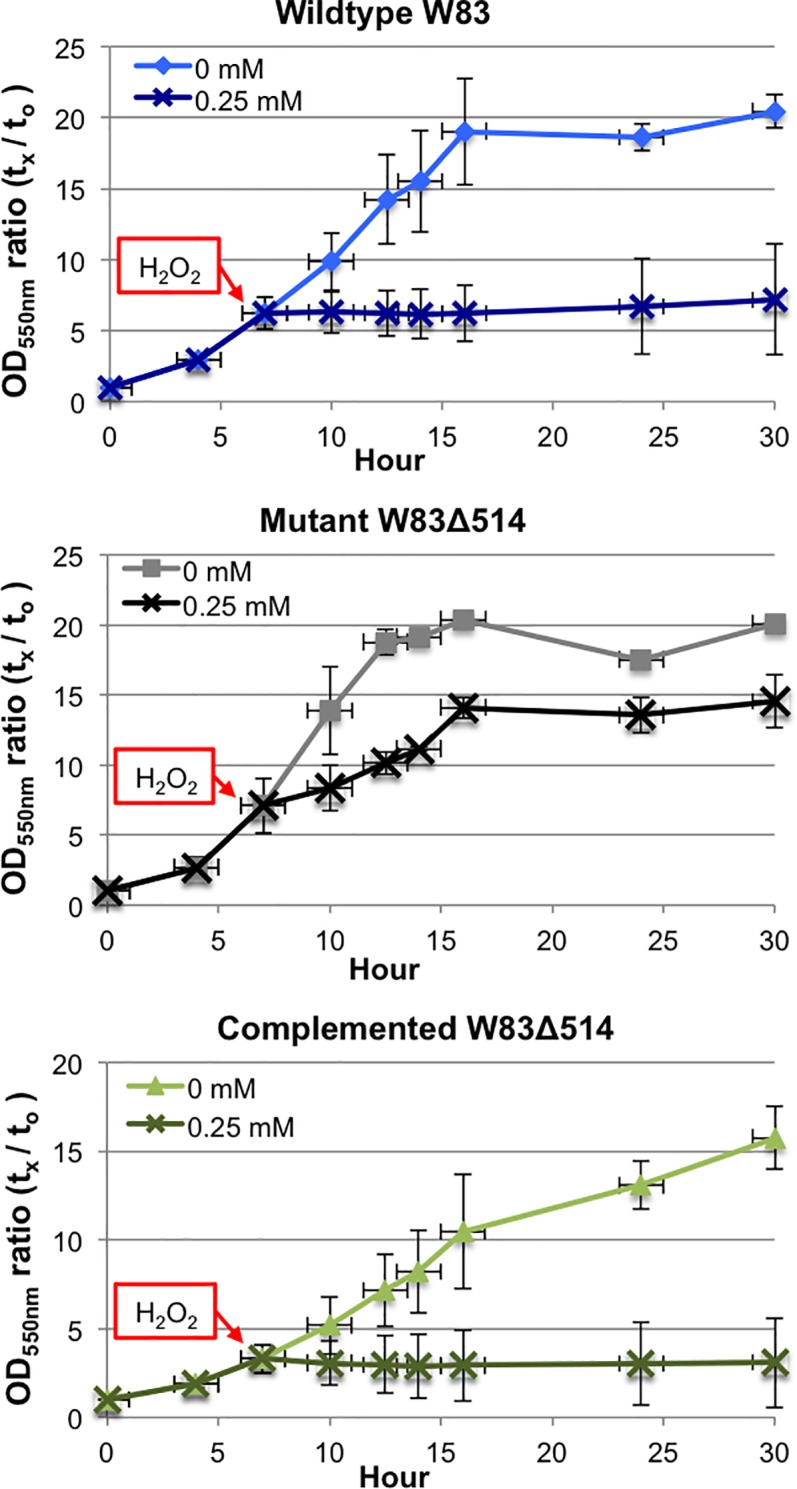
Complementation of W83Δ514 containing a chromosomal insertion of PG_RS02100 restored hydrogen peroxide sensitivity. Pre-reduced HL-media were inoculated (starting OD_550nm_ = 0.16–0.17) with bacteria from HL-stationary-cultures. H_2_O_2_ was added when the 10 ml cultures reached early log-phase (OD_550nm_ = 0.5–0.7) to a final concentration of 0.25 mM and the optical density was recorded at the specified time points over 30 h. Data is reported as a ratio of average OD (t_x_) to average starting OD (t_0_) ± SD.

After plating on enriched BAP, W83Δ514 was more resistant to continuous atmospheric dioxygen exposure compared to W83 (Fig 7) and was more resistant to hydrogen peroxide stress when cultured under HL but not under HR growth conditions (Fig 8). The results with exposure to 0.25 mM H_2_O_2_ were confirmed in four separate experiments (Fig 8A and 8B). Additional experiments that assessed a range of H_2_O_2_ concentrations (0.125 mM, 0.25 mM, 0.5 mM) also supported these findings but only the data after 0.5mM exposure is shown; W83Δ514 was more resistant to 1h 0.5 mM H_2_O_2_ exposure under HL growth conditions (Fig 8C) but no difference was observed relative to the wildtype under HR growth conditions. Complementation in the mutant restored hydrogen peroxide sensitivity (Fig 9).

### Comparison of the relative expression levels of select genes linked to oxidative stress survival between mutant and wildtype strains

Quantitative RT-PCR expression analysis was performed using total RNA isolated from W83Δ514 and W83 from six culture and/or treatment conditions: 1) Log phase under HR anaerobic conditions, 2) Log phase under HL anaerobic conditions, 3) Log phase under HR anaerobic conditions after 1 h hydrogen peroxide exposure (0.25 mM H_2_O_2_), 4) Log phase under HL anaerobic conditions after 1 h hydrogen peroxide exposure (0.25 mM H_2_O_2_), 5) Log phase under HR anaerobic conditions after 1 h atmospheric dioxygen exposure with shaking, and 6) overnight HL anaerobic cultures passaged daily with fresh modified TSB media without hemin for 2-days reported as 2-day HS. The relative expression of *oxyR*, *trx-1*, *tpx*, *sodB*, *ahpC*, *dinF*, *cydB*, and *frd* in the mutant compared to the wildtype were expressed as relative fold change with negative values indicating decreased expression in the mutant (Table 1).

W83Δ514 displayed 5.13 fold reduction in *oxyR* expression under heme rich conditions but a 4.41 fold higher expression of *oxyR* under anaerobic heme starvation, suggesting that constitutive expression of *oxyR* is linked to heme availability and that this association is altered in the mutant. Expression of *oxyR* was similar in the mutant relative to the wildtype when exposed to exogenous hydrogen peroxide and atmospheric dioxygen, which is consistent with studies that report that *oxyR* expression is not induced in *P*. *gingivalis* under these conditions [17, 18].

*Trx-1* expression was similar in the W83Δ514 relative to wildtype during anaerobic growth under HR or HL conditions. In contrast, expression of *trx-1* was 12.30 fold higher after 2-day HS in W83Δ514 relative to W83. After 1 h exposure to exogenous hydrogen peroxide, expression of *trx-1* was higher in the mutant than the wildtype in a heme (increasing) dependent manner, shifting from 2.51 to 4.21 fold higher under HL and HR conditions respectively. In contrast, no notable difference in expression of *trx-1* between mutant and wildtype was observed after 1 h atmospheric dioxygen exposure.

D*ps* expression in W83Δ514 was no different than W83 under HR conditions, but it was 3.36 fold higher than wildtype under anaerobic HL conditions. This higher expression disappeared under 2-day HS condition. There was no difference in the expression of *dps* between W83Δ514 and wildtype following hydrogen peroxide exposure. This observation is consistent with published reports that *dps* expression increases with increased heme availability [19]. However, *dps* expression was -4.94 fold lower in W83Δ514 after 1 h exposure to atmospheric dioxygen.

**Fig 10:**
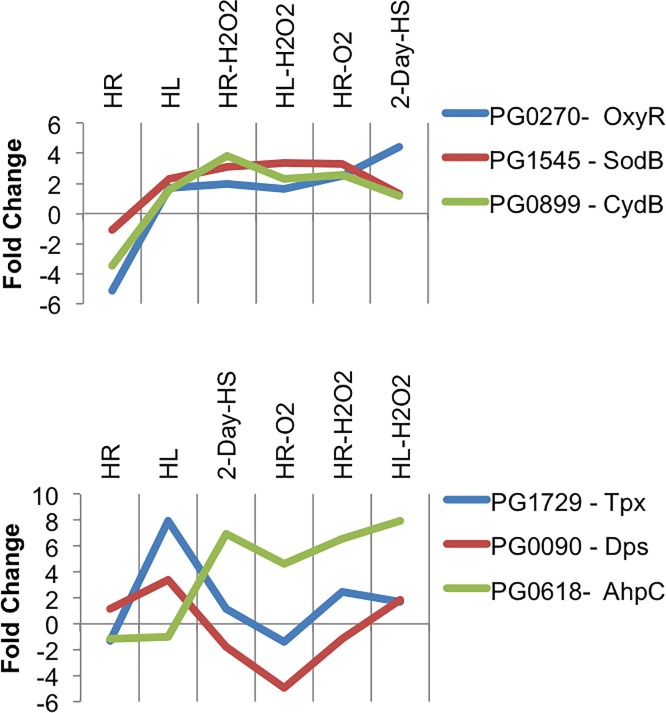
Comparison of relative qPCR expression profiles. The average fold-change in expression of specified gene targets involved in oxidative stress in W83Δ514 mutant relative to expression in wildtype strain W83 under six different culture and/or oxidative stress conditions were plotted to visualize their relative expression profiles.

Overall, *tpx* expression in W83Δ514 was similar to W83 with the exception of growth under HL whereby *tpx* expression was 7.96 fold higher. Overall, the relative expression profile of *dps* correlates with the *tpx* expression profile (Fig 10). Seemingly in conflict, the expression of *tpx* and dps after 2-day HS was similar between W83Δ514 and W83 despite higher relative expression of their transcriptional regulator *oxyR* [20] in W83Δ514 relative to W83.

Though expression of *cydB* was -3.47 fold lower under anaerobic HR conditions and 3.84 fold higher after 1 h exposure to hydrogen peroxide under HR conditions in W83Δ514 relative to the wildtype, expression of *sodB* was over 3 fold higher when under oxidative stress or after 2-day HS stress in W83Δ514. When assessed broadly across all the conditions tested, the relative expression profile of *cydB* correlates with the expression profile of the antioxidant enzyme *sodB* (Fig 10). Moreover, the relative expression profile of *sodB* in W83Δ514 correlates with the expression profile of *oxyR*, its transcriptional regulator [21], under all conditions except after 2-day HS, much like *tpx* and *dps* (Fig 10).

W83Δ514 *ahpC* expression was similar to W83 when grown under HR or HL conditions, but a*hpC* expression increased 6.96 fold when bacteria were grown under 2-day anaerobic heme starvation. In contrast, expression of *ahpC* was 6.59 and 7.96 fold higher in W83Δ514 in response to 1 h exposure to peroxide, under HR or HL conditions respectively, relative to wildtype. W83Δ514 *ahpC* expression also increased 4.59 fold in response to dioxygen. Interestingly, though ahpC expression is reportedly also regulated by OxyR in *P*. *gingivalis* [17], as with *sodB*, *dps* and *tpx*, its overall expression profile demonstrated a much higher expression in W83Δ514 under all exogenous oxidative stress conditions and after 2 day heme starvation but similar expression under anaerobic, presumably non-stressed, growth compared to wildtype (Fig 10).

Irrespective of growth conditions, expression of *dinF* and *frdB* in W83Δ514 was similar to that in W83. In contrast, expression of *dinF* was 4.69 fold higher in W83Δ514 after 1 h atmospheric dioxygen exposure.

## Discussion

*Porphyromonas gingivalis* is recognized as a major pathogen of adult periodontitis and implicated in multiple systemic inflammatory conditions including cardiovascular disease. The ability of *P*. *gingivalis* to invade a wide variety of host cell types and employ adaptive mechanisms to subvert specific host responses are key virulence factors critical to its survival and persistence. However, unlike other bacteria such as *E*. *coli*, analysis of the genome indicates that *P*. *gingivalis* lacks the RNA binding protein Hfq, which facilitates post-transcriptional gene regulation during stress responses [22]. We previously identified putative sRNAs using both NimbleGen microarray analysis and Illumina sequencing in *P*. *gingivalis* [8]. In this study we characterized an IGR encoded transcript, PG RS02100 that we originally identified as being significantly expressed during log-phase, which appears to be linked to processes shown to modulate *P*. *gingivalis* virulence.

Though not reported in our results, when we first identified this transcript as a possible regulatory sRNA [8], during our preliminary *in silico* analysis we had used the online tool targetRNA2 [23] to predict sRNA binding targets based on the reported success of this predictive program. The predicted transcripts of *dprA* (PG0295 / PG_RS01330), *aroC* (PG1314 / PG_RS05785), and *cydB* (PG0899 / PG_RS03960) were identified as potential mRNA binding targets of PG_RS02100 that would most energetically favor binding the region overlapping the first 70nt of this transcript (P-value <0.003). We had hypothesized at that time that if this transcript indeed functions as a regulatory sRNA, and binds to one or more of these hypothetical targets, PG_RS02100 may function to adapt to oxygen stress and /or metabolism, and thus we sought to assess these characteristics during our analyses reported in this study. Without demonstrating direct binding however, we have no evidence that PG_RS02100 is a regulatory sRNA. It is possible that it was serendipity that our observations support our early *in silico* phenotypic predictions. Similarly, the identification of a putative open reading frame, particularly one with a rare *E*. *coli* start codon that has yet to be demonstrated as functional in *P*. *gingivalis*, does not prove a functional protein is produced from PG_RS02100. This study only demonstrates that knocking out expression of the PG_RS02100 RNA transcript alters *P*. *gingivalis* phenotype as described here using a wide variety of assays. In our future work, we plan to determine PG_RS02100 mode of action.

Our studies to date have been focused on phenotypically characterizing this putative transcript PG_RS02100 by comparative analysis of the deletion mutant W83Δ514 to the wildtype strain with the aim of generating evidence of its biological relevance. PG_RS02100 was differentially expressed relative to growth phase, with expression observed during mid-log but not stationary phase of growth, irrespective of heme availability. Under standard anaerobic conditions, irrespective of heme availability, our deletion mutant of PG_RS02100 (W83Δ514) had the same growth rate as wildtype W83.

Colony pigmentation is caused by accumulation of μ-oxo heme dimer on the cell surface. We found that our mutant strain acquired pigmentation more rapidly than the wildtype when cultured on whole blood agar plates but not laked blood agar plates, suggesting that the mutant had an advantage in acquiring hemin from non-lysed whole blood cells. To the best of our knowledge, this is the first report of hyperpigmentation observed in *P*. *gingivalis*. Colonial pigmentation on blood agar plates has been shown to be linked with activities of major proteinases, Arg-gingipain (Rgp) and Lys-gingipain (Kgp), and other virulence factors, suggesting that colonial pigmentation is associated with the presence of gingipain-adhesin complexes on the cell surface [24]. Interestingly, our data suggest that under heme rich conditions, stationary broth cultures of the mutant had decreased gingipain activity compared to the wildtype, which is consistent with previous studies that have shown that kgp and rgpA expression is regulated by growth phase and not heme availability [25].

As an obligate anaerobe and a keystone periodontal pathogen that commonly colonizes most surfaces in the oral cavity, *P*. *gingivalis* must be able to resist exogenous oxidative stress. It is widely understood that *P*. *gingivalis* preferentially colonizes the periodontal sulcus and grows in concert with other oral microorganisms in a biofilm, presumably in response to dioxygen levels. It is also widely understood that periodontal inflammation in response to oral biofilm leads to host mediated production of elevated levels of reactive oxygen species (ROS) with collateral tissue damage. More recently, studies have reported that serum reactive oxygen metabolites (ROMs) are also elevated in patients with periodontitis [26, 27]. Thus, the ability of *P*. *gingivalis* to disseminate and persist in the diseased host environment appears to necessitate its ability to survive oxidative stress. *P*. *gingivalis* is known to produce multiple factors to protect itself from oxidative stress, whether growing extracellularly in oral biofilms or intracellularly.

In many microorganisms, OxyR is a redox-sensitive transcriptional activator that plays a role in surviving exogenous hydrogen peroxide, and for some organisms, play a role in atmospheric dioxygen tolerance [20]. In these organisms, OxyR is typically activated in the presence of exogenous hydrogen peroxide, inducing the expression of genes it regulates [17]. However, in *P*. *gingivalis*, OxyR, does not induce *ahpCF*, *dps*, and *sodB* expression in response to hydrogen peroxide despite the fact that the expression of these oxidative-stress-related genes still require OxyR, albeit under anaerobic conditions [17, 18]. In other words, *oxyR* appears to be constitutively expressed under anaerobic conditions in *P*. *gingivalis*. It is suggested that *P*. *gingivalis* OxyR may also play a partial role in aerotolerance but its expression is not induced during exposure to atmospheric oxygen [17, 28], nor microaerophilic conditions [29]. Our expression data is consistent with the suggestion that other regulators besides *oxyR* play a role in sensing and protecting *P*. *gingivalis* against exogenous dioxygen stress. Moreover, when comparing the expression profile of *oxyR* in W83Δ514 relative to the W83 across all conditions assayed, it appears that the constitutive expression of *oxyR* may be slightly higher in the more aerotolerant W83Δ514.

For an obligate anaerobe where protection against exogenous hydrogen peroxide and atmospheric dioxygen is arguably more critical, a primed oxidative stress response system that constitutively expresses OxyR and that responds at the post-transcriptional or post-translational level would be advantageous. In *P*. *gingivalis*, a study showed that OxyR protein is activated in response to heme limitation but *oxyR* gene expression was not altered [30]. Notably, *P*. *gingivalis* OxyR activity is dependent on an intramolecular disulfide bond that is cleavage regulated (deactivated) by thioredoxin, and thioredoxin-1 expression is induced in the presence of atmospheric dioxygen [17, 31]. Mutation of thioredoxin-1 results in increased tolerance to exogenous hydrogen peroxide in *P*. *gingivalis* [30]. Moreover, expression of thioredoxin-1 (*trx-1*) was also reported to be reduced, along with *sodB*, *dps*, and *ahpC*, in response to decreased heme availability (limited and starved) [30], but it does not appear to be regulated by OxyR like other thioredoxins. For example, expression of thioredoxin-2 (PG_RS05035 / PG1134), and especially thioredoxin-like protein (PG_RS01230 / PG0275), is reportedly reduced in an *oxyR* mutant of *P*. *gingivalis* [17, 30] but little to no change in expression of *trx-1* (PG_RS00170 / PG0034) was observed [30].

In *P*. *gingivalis*, it appears that the bacterium's response to heme availability is linked both directly and indirectly to oxidative stress tolerance in a complex manner. However, there are many gaps in our knowledge as to how these systems interact and which gene products are involved. Although 2-day heme starvation enhanced expression of *trx-1*, *oxyR*, and *ahpC* in W83Δ514, expression of *dps*, *sodB*, and *tpx* were essentially unchanged under this condition, indicating it is unlikely that oxidative stress from some indeterminate exogenous source occurred. As stated previously, studies show that expression of *trx-1* increases when heme availability increases, seeming to reflect an increased dependence on thioredoxins like *trx-1* to maintain a reduced state in *P*. *gingivalis*. In context of our other observations, the higher expression of trx-1 observed after hydrogen peroxide exposure in W83Δ514 relative to W83 likely reflects an altered response to heme availability in the mutant due to its altered ability to accumulate heme, however, we cannot rule out that there was hydrogen peroxide mediated induction of *trx-1* expression in W83Δ514. Moreover, our data suggests that Trx-1 may play a role in iron starvation induced stress response in *P*. *gingivalis*.

Dps is a starvation/stationary phase-inducible DNA-binding protein involved in protection against hydrogen peroxide in *E*. *coli* and *B*. *fragilis* [32, 33]. In *P*. *gingivalis*, *dps* expression is reportedly not positively regulated in response to exogenous hydrogen peroxide [17] although one study reported a slight induction of *dps* expression after exposure to lethal levels of hydrogen peroxide (0.5 mM) [18]. Another study reported that Dps binds to heme in *P*. *gingivalis* and has been shown to be involved in tolerance to heme toxicity, indirectly protecting DNA from hydrogen peroxide mediate damage [19]. Consequently, a *dps* mutant is sensitive to exogenous hydrogen peroxide under heme excess [19]. *Dps* expression reportedly increases with greater heme availability [19] and its expression is induced in the presence of atmospheric dioxygen in *P*. *gingivalis* [28]. Our observations suggest that W83Δ514 can bind heme more readily on whole blood agar plates during early colonization and growth, which would lead to altered sensitivity to heme availability, and thus it is not unexpected that the expression profile of *dps* in W83Δ514 was also be altered compared to wildtype. Notably, *dps* expression was much lower in W83Δ514 after 1 h exposure to atmospheric dioxygen. It seems highly unlikely that *dps* expression is being down regulated in W83Δ514 in response to atmospheric dioxygen, but rather it is not as strongly induced in the mutant as observed in the more oxidative stress sensitive W83.

The impact of deleting PG RS02100 on expression of *tpx*, *sodB*, and *ahpC*, was investigated because they encode key antioxidant enzymes. Thiol peroxidase (Tpx) is involved in protecting the cell from atmospheric dioxygen and hydrogen peroxide, is regulated by OxyR, and functionally linked to thioredoxins in *B*. *fragilis* [20, 28, 34]. In *P*. *gingivalis*, expression of *tpx* is reportedly induced in the presence of atmospheric dioxygen and regulated by OxyR [28]. Superoxide dismutase (Fe-Mn) (SodB) has been shown to be regulated by OxyR, atypical of most identified microbial SodB homologs [17, 21], and is reportedly regulated in a heme dependent manner in *P*. *gingivalis* [30]. Alkyl hydroperoxide reductase subunit C (AhpC) protects against organic peroxides [35], and is regulated by OxyR in *P*. *gingivalis* [17]. The elevated relative expression of *sodB* and *ahpC* in W83Δ514 under oxidative stress conditions are consistent with our phenotypic observation of greater aerotolerance of W83Δ514 under continuous atmospheric dioxygen exposure and survival after hydrogen peroxide exposure under HL conditions. In contrast, the expression profile of *tpx* indicates that its expression is impacted by heme availability, rather than a significantly altered response to hydrogen peroxide exposure in the mutant, reflective of its altered ability to bind heme. While this does not obviously support our phenotypic observation that W83Δ514 is better able to survive hydrogen peroxide exposure under HL but not HR conditions relative to the wildtype, it does support the link between tolerance to oxidative stress and heme availability / toxicity in *P*. *gingivalis*.

As stated previously the relative expression profile of *tpx* and *sodS*, along with *dps*, was generally consistent with the *oxyR* expression profile except after 2-day HS, where their expression was not as high as would have predicted based strictly on the relative *oxyR* expression in W83Δ514. This would only make sense if *oxyR* expression were not indicative of OxyR activity in W83Δ514 under this condition. This is possible if one considers the 12.30 fold higher relative expression of *trx-1* in W83Δ514 observed after 2-day HS, which may have led to inactivation of OxyR. In contrast, *ahpC* expression was much higher in W83Δ514 compared to the wildtype when heme starved for 2-days under anaerobic growth conditions. A possible explanation of this single exception, compared to the expression of the other OxyR regulated genes, is that *ahpC* expression may still be induced at lower active OxyR concentrations than that required for the other genes. This possibility is supported by a study that reported *ahpC* expression decrease 16.3 fold in an *oxyR* mutant, while expression of *dps* and *sodB* were only reduced 6.70 and 5.73 fold respectively in an *oxyR* mutant during anaerobic growth in *P*. *gingivalis* [17]. Alternatively, the *ahpC* expression profile had a much higher relative expression in W83Δ514 under all exogenous oxidative stress conditions as well as after 2-day heme starvation, suggesting that *ahpC* expression is likely modulated by a stress sensor in *P*. *gingivalis* and that this hypothetical sensor is more responsive in W83Δ514 (Fig 10).

DinF is an integral membrane protein with the predicted annotation of DNA-damage-inducible protein F in *P*. *gingivalis*. In *E*. *coli*, *dinF* expression is regulated as part of the SOS system and has been shown to provide protection against oxidative stress, including tolerance to exogenous hydrogen peroxide, through a yet unidentified mechanism [36, 37]. As stated previously, expression of *dinF* was much higher in W83Δ514 after 1 h atmospheric dioxygen exposure, suggesting that DNA in W83Δ514 would likely be better protected from oxygen than in the wildtype. This data is consistent with W83Δ514 being better able to survive atmospheric dioxygen exposure than the wildtype when plated on enriched BAP (Fig 6). However, it is unclear mechanistically why W83Δ514 was more responsive than the wildtype resulting in greater induction of *dinF*.

In facultative anaerobes such as *E*. *coli* or anaerobes that undergo anaerobic respiration such as *B*. *fragilis*, cytochrome ubiquinol oxidase, made up of heme-b cofactor /subunit-I (CydA) and heme-d / subunit-II (CydB), plays a role in energy metabolism under oxygen limiting conditions due to its very high affinity for oxygen relative to other oxidases found in these organisms [38]. This oxidase has catalase activity, functioning to neutralize hydrogen peroxide in these organisms [38]. In *P*. *gingivalis*, cydAB expression is reported to be induced in response to exogenous atmospheric dioxygen [29] and is important for tolerance to atmospheric dioxygen and exogenous hydrogen peroxide during exponential growth [38]. Similarly, SodB plays a role in protecting *P*. *gingivalis* from atmospheric dioxygen [39] and its expression is reported to be induced in response to hydrogen peroxide in a dose dependent manner in *P*. *gingivalis* [18]. As stated previously, the relative expression profile of *cydB* in W83Δ514 correlates with the expression profile of *sodB*, suggesting they respond to exogenous oxidative stress and heme availability similarly.

While our data supports the premise that CydB functions to scavenge exogenous dioxygen, rather than enzymatically neutralizing dioxygen or ROS like SodB, it has been hypothesized that CydB may also play some role in metabolism by diminishing the rate of ROS generation mediated by fumarate reductase (Frd) as observed in other bacterial species when growing under anaerobic conditions [29]. For this reason, we also assayed the expression level of *frdB* (iron-sulfur subunit) to ascertain if our mutant can reveal a link between CydB and FrdB despite a study reporting that expression of *frdB* is unaffected by microaerophilic conditions in *P*. *gingivalis* [29]. We could not ascertain any such link based on our data. The expression of *frdB* in W83Δ514 was similar to wildtype under all conditions tested, supporting the observation that there was no change in the growth curve between W83Δ514 and W83. Reports suggest that *P*. *gingivalis* may possibly undergo fumarate respiration under anaerobic or microaerophilic conditions during biofilm development through fumarate reductase (Frd) [29, 38, 40, 41], likely linked to heme availability and the activity of redox modulating enzymes to neutralize ROS generated by Frd, but more studies are needed to support this hypothesis.

## Conclusions

Based on the expression profile of the genes reported in this study, selected due to their direct or indirect association with oxidative stress, and the pleiotropic phenotype resulting from deletion of PG_RS02100, we hypothesize that this transcript modulates *P*. *gingivalis* survival under stress conditions. Moreover, based on data collected from our cultural characterization of W83Δ514, our hypothesis is that PG_RS02100 plays a role, directly or indirectly, in cell surface composition, especially the acquisition of heme.

Future studies will investigate the contribution of PG_RS02100 in intracellular survival in which the nutritional conditions can vary significantly, depending on the length of infection and the host cell type. We also intend to investigate whether or not other phenotypic changes have occurred associated with the cell surface, such as its ability to form biofilm and excrete capsule polymers, which would impact *P*. *gingivalis’* ability to colonize the oral cavity and survive environmental stress.

## Supporting information

S1 Table Primers used to create mutant W83-Δ514 and the complement strains. (DOCX)

S2 Table Primers used to of qRT-PCR comparative expression analysis. (DOCX)

S3 Table Primers used to detect expression of PG_RS02100. (DOCX)

S4 Table Intergenic region, between the PG_RS02095 and PG_RS02105 TAA ochre stop codons, that codes for the PG_RS02100 transcript. The start (green) and stop (red) codons are boxed. The following features are denoted in the sequence as described: The 102nt sequence that was deleted and replaced with *Erm*^*R*^ gene in bold and italicized (NC_002950.2:514663–514765). The 429nt from the illumina sequenced 5’-end of the PG_RS02100 transcript [8] (indicated by a forward slash interrupting the sequence), to the end of predicted Rho-independent terminator (underlined sequence) that may code for a regulatory small RNA (sRNA). The putative coding sequence of PG_RS02100 from the predicted rare start codon TTG (that typically codes for a leucine) to the opal stop codon TGA (W83 reference genome loci: NC_002950.2:514604–515005).
(DOCX)

S5 Table Nucleotide sequenced spanning the IGR between the PG_RS02095 and PG_RS02105 TAA ochre stop codons in the mutants W83Δ514 and W83Δ514-Complement strains. The start (green) and stop (red) codons are boxed. The following features are denoted in the sequence as described: The sequence containing the expressible *ErmF*(PG) / *ErmAM*(E.coli) cassette is italicized. The sequence containing the expressible *TetQ* gene is underlined and in opposite orientation to the *ErmF* gene. The completely restored intergenic region in the complement strain that codes for the PG_RS02100 sequence is in bold.
(DOCX)

S1 Appendix Gingipain assay. (DOCX)

S1 Fig BLAST and pfam taxonomy analysis output (Query IDs WP_012458318.1 and DUF4248 respectively). (TIFF)
