# Supplementary material for: Deletion of a conserved transcript PG_RS02100 expressed during logarithmic growth in Porphyromonas gingivalis results in hyperpigmentation and increased tolerance to oxidative stress
Source: PLoS One. 2018 Nov 12;13(11):e0207295. doi: 10.1371/journal.pone.0207295 (PMC6231650; doi:10.1371/journal.pone.0207295)
Supplement: S1 Table — (DOCX) [file pone.0207295.s001.docx]

**S1 Table. Primers used to create mutant W83-Δ514 and the complement strains.**

| **Template** | **ID** | **5’ – 3’ sequence*** | **PCR product** | **Product description** |
| --- | --- | --- | --- | --- |
| W83 | 514663UF | TTCGTCGGGAGGTCTGCCCC | 0.79 kb | Upstream sequence to gene target PG_RS02100 |
|  | 514663UR/E | AGCGGAAGCTATCGGGGGTACCACATTTCGCACCGATCTGCCG |  |  |
| W83 | 514663DF/E | ATCCTCTAGAGTCGACCTGCAGTCTAACGGCGCATCGGCACG | 0.79 bp | Downstream sequence to gene target PG_RS02100 |
|  | 514663DR | ATCGGCGGCACCAAGAACGG |  |  |
| pTCow/Erm | Erm514663F | AAATGTGGTACCCCCGATAGCTTC | 2.19 kb | Erm cassette, mutant selectable marker |
|  | Erm514663R | GTTAGACTGCAGGTCGACTCTAGAGG |  |  |
| Fusion PCR product | 514663NF | GCCTCCCGATGCTCCGATGC | 3.47 kb | Nested PCR product for transformation of W83 to generate deletion mutant |
|  | 514663NR | CAAAGGGCGCGTGCTGGGTA |  |  |
| W83-Δ514 Mutant | 514663C_F1_Fw | CGCATAACGGCTGGCAA | 0.97 kb | Upstream sequence in the mutant, containing the Erm cassette |
|  | 514663C/tetQ_F1_Rv | ATGGAGCGGTCATTCCCTTTAGTAACGTGTA |  |  |
| pTCow/tetQ | 514663C/tetQ_F2_Fw | AGGGAATGACCGCTCCATTATTTTGATG | 2.47 kb | TetQ cassette, complement selectable marker |
|  | 514663C/tetQ_F2_Rv | *CCGTT*GGCCCTCAAACCCCG |  |  |
| W83 | 514663C/TetQ_F3_Fw | *AGGGCCAACGGTTCAAATTACGAATGC* | 1.71 kb | Complete PG_RS02100 and downstream sequence to restore 514 |
|  | 514663C_F3_Rv | *CATTTCCGGCACAAGATCAATAC* |  |  |
| Fusion PCR product | 514663C_NF | GCGATGGAGCGGAAACGTA | 5.08 kb | Nested PCR product for transformation of mutant to generate complement |
|  | 514663C_NR | AACATAGCTTATGAGCAAGATCAGC |  |  |
| *Underscore indicates Erm cassette sequence. Italics indicates TetQ cassette sequence. | | | | |
