# Supplementary material for: Deletion of a conserved transcript PG_RS02100 expressed during logarithmic growth in Porphyromonas gingivalis results in hyperpigmentation and increased tolerance to oxidative stress
Source: PLoS One. 2018 Nov 12;13(11):e0207295. doi: 10.1371/journal.pone.0207295 (PMC6231650; doi:10.1371/journal.pone.0207295)
Supplement: S2 Table — (DOCX) [file pone.0207295.s002.docx]

**S2 Table. Primers used to of qRT-PCR comparative expression analysis.**

| Annotation | Primer ID | Gene Locus | 5’ – 3’ Sequence |
| --- | --- | --- | --- |
| 16S ribosomal RNA | PG16SrRNAF | PG_RS05595; PG_RS09195; PG_RS00460; PG_RS07365 | AAACCGTCTTCCCTTCGGG |
|  | PG16SrRNAR |  | TGTAAGGGCCGTGCTGATTT |
| cydB, cytochrome d ubiquinol oxidase, subunit II | PG0899F | PG_RS03960 | ACGGCAAATCCTTCCGTAGT |
|  | PG0899R |  | TTGAACCTGTGGAACGTGGT |
| OxyR transcriptional regulator | PG0270F | PG_RS01210 | TTGCTTCAGAGATCAGCTCGT |
|  | PG0270R |  | GACGAGGCATACCAAACGGA |
| Superoxide dismutase (Mn/Fe) | PG1545F | PG_RS06820 | AAACTGGGGGAAGCTATCGAC |
|  | PG1545R |  | ACGTCGAATCCGAGCAAAGG |
| Dps, DNA protection /binding protein of starved cells | PG0090F | PG_RS00405 | TCAGCGAGTACCTGAAAGTAGC |
|  | PG0090R |  | AACGAGCTTCTCTTGCTCGG |
| DinF, DNA damage inducible protein F | PG1640F | PG_RS07215 | CTGCCCAGAAAGCAAAACGG |
|  | PG1640R |  | CGAAGCACTTACGGGACGAT |
| ahpC, alkyl hydroperoxide reductase | PG0618F | PG_RS02725 | TATCCCATGTTGGCCGATCC |
|  | PG0618R |  | GCAGCTACGTATTGTGCAGC |
| Trx, thioredoxin | PG0034F | PG_RS00170 | ACCACTTCGCCGTTCTTGAT |
|  | PG0034r |  | AAGCCGATGGTAGTGGATTTCT |
| Tpx, thiol peroxidase | PG1729F | PG_RS07610 | AGTGATCTCGGGAACAAGCTC |
|  | PG1729R |  | CTTTTGCACAGGCTCGCTTC |
| FrdB, fumerate reductase | PG1614F | PG_RS07115 | TCCCTATTCAGACGGGCAATG |
|  | PG1614R |  | TCGGCTATGCTCTTCGTATCG |
