# Supplementary material for: Deletion of a conserved transcript PG_RS02100 expressed during logarithmic growth in Porphyromonas gingivalis results in hyperpigmentation and increased tolerance to oxidative stress
Source: PLoS One. 2018 Nov 12;13(11):e0207295. doi: 10.1371/journal.pone.0207295 (PMC6231650; doi:10.1371/journal.pone.0207295)
Supplement: S3 Table — (DOCX) [file pone.0207295.s003.docx]

**S3 Table. Primers used to detect expression of PG_RS02100.**

| Gene locus / Primer ID | 5’ – 3’ Sequence |
| --- | --- |
| PG_RS02100R | AGAATAATGATGAATACCGAACAGG |
| PG_RS02100F | GCAGGTGTAGCGATGCATTT |
