# Supplementary material for: Deletion of a conserved transcript PG_RS02100 expressed during logarithmic growth in Porphyromonas gingivalis results in hyperpigmentation and increased tolerance to oxidative stress
Source: PLoS One. 2018 Nov 12;13(11):e0207295. doi: 10.1371/journal.pone.0207295 (PMC6231650; doi:10.1371/journal.pone.0207295)
Supplement: S4 Table — The start (green) and stop (red) codons are boxed. The following features are denoted in the sequence as described: The 102nt sequence that was deleted and replaced with ErmR gene in bold and italicized (NC_002950.2:514663–514765). The 429nt from the illumina sequenced 5’-end of the PG_RS02100 transcript [8] (indicated by a forward slash interrupting the sequence), to the end of predicted Rho-independent terminator (underlined sequence) that may code for a regulatory small RNA (sRNA). The putative coding sequence of PG_RS02100 from the predicted rare start codon TTG (that typically codes for a leucine) to the opal stop codon TGA (W83 reference genome loci: NC_002950.2:514604–515005). (DOCX) [file pone.0207295.s004.docx]

**S4 Table. Intergenic region, between the PG_RS02095 and PG_RS02105 TAA ochre stop codons, that codes for the PG_RS02100 transcript.**

| TAACGGTTCAAATTACGAATGCCCTTGGGGAAAACGATTCCTTTCTCTTCTCGAATCGTTGACTTTTCTGGTTATTCTGATTACTTAGATTCTTCCGATGCATAGTCGAAAAGATAAATATAGCCTTCACTG**TTG**ATAACTCGGTGCGAAAAGTATCGGCAGATCGGTGCGAAATGT***ATTCCTTTGTATTT/GTCTGCCGGCAGGTGTAGCGATGCATTTTTCAGCCTGTTCGGTATTCATCATTATTCTATAAAAACACATACCAACATGAAAGTTAAA***TCTAACGGCGCATCGGCACGGGAGGATGTATTCCCATTCCGAGTCCATCGCGTCAAAGAAGTAGCCGTGCGCTACTTTCCGCACCTTACTCCGAATTCCGGTACACGTGCTCTGCGAAGAATCATCTATGGGGATCAAGATCTCCTCAATTCGATGAGAGAACATGGTTATGCTCTGGGACAGAGATCCTTGACACCGGCTATGCTCAACGTCCTTACAGCCTATCTTGGTTCGCCCGAAGATTTTTGTCCTTGACGGACAATAGCTGAAAGTCTGCTTATAGGGGGTATGTCGAAGTCGTGATTTGCGGCTTTCGGCATACCCCCTTTCAGTATATGCAAAGAGGATGTTTA |
| --- |

The start (green) and stop (red) codons are boxed. The following features are denoted in the sequence as described: The 102nt sequence that was deleted and replaced with *Erm^R^* gene in bold and italicized (NC_002950.2:514663-514765). The 429nt from the illumina sequenced 5’-end of the PG_RS02100 transcript [1] (indicated by a forward slash interrupting the sequence), to the end of predicted Rho-independent terminator (underlined sequence) that may code for a regulatory small RNA (sRNA). The putative coding sequence of PG_RS02100 from the predicted rare start codon TTG (that typically codes for a leucine) to the opal stop codon TGA (W83 reference genome loci: NC_002950.2:514604-515005).

References

1. Phillips P, Progulske-Fox A, Grieshaber S, Grieshaber N. Expression of Porphyromonas gingivalis small RNA in response to hemin availability identified using microarray and RNA-seq analysis. FEMS microbiology letters. 2014;351(2):202-8. doi: 10.1111/1574-6968.12320. PubMed PMID: 24245974; PubMed Central PMCID: PMCPMC4009720.
