# Supplementary material for: Deletion of a conserved transcript PG_RS02100 expressed during logarithmic growth in Porphyromonas gingivalis results in hyperpigmentation and increased tolerance to oxidative stress
Source: PLoS One. 2018 Nov 12;13(11):e0207295. doi: 10.1371/journal.pone.0207295 (PMC6231650; doi:10.1371/journal.pone.0207295)
Supplement: S5 Table — The start (green) and stop (red) codons are boxed. The following features are denoted in the sequence as described: The sequence containing the expressible ErmF(PG) / ErmAM(E.coli) cassette is italicized. The sequence containing the expressible TetQ gene is underlined and in opposite orientation to the ErmF gene. The completely restored intergenic region in the complement strain that codes for the PG_RS02100 sequence is in bold. (DOCX) [file pone.0207295.s005.docx]

**S5 Table. Nucleotide sequenced spanning the IGR between the PG_RS02095 and PG_RS02105 TAA ochre stop codons in the mutants W83∆514**

**and W83∆514-Complement strains.**

| W83∆514 sequence |
| --- |
| TAACGGTTCAAATTACGAATGCCCTTGGGGAAAACGATTCCTTTCTCTTCTCGAATCGTTGACTTTTCTGGTTATTCTGATTACTTAGATTCTTCCGATGCATAGTCGAAAAGATAAATATAGCCTTCACTGTTGATAACTCGGTGCGAAAAGTATCGGCAGATCGGTGCGAAATGT*ggtacccccgatagcttccgctattgcttttttgctcatcggtatttgcaacatcatagaaattgcatacctttgttcctcggttatatgtttgctcatctgcaacttttttttctttggacggacaattaaagcaaagatagcaaactttatccattcagagtgagagaaagggggacattgtctctctttcctctctgaaaaataaatgtttttattgcttattatccgcacccaaaaagttgcatttataagttgaactcaagaagtattcacctgtaagaagttactaatgacaaaaaagaaattgcccgttcgttttacgggtcagcactttactattgataaagtgctaataaaagatgcaataagacaagcaaatataagtaatcaggatacggttttagatattggggcaggcaaggggtttcttactgttcatttattaaaaatcgccaacaatgttgttgctattgaaaacgacacagctttggttgaacatttacgaaaattattttctgatgcccgaaatgttcaagttgtcggttgtgattttaggaattttgcagttccgaaatttcctttcaaagtggtgtcaaatattccttatggcattacttccgatattttcaaaatcctgatgtttgagagtcttggaaattttctgggaggttccattgtccttcaattagaacctacacaaaagttattttcgaggaagctttacaatccatataccgttttctatcatactttttttgatttgaaacttgtctatgaggtaggtcctgaaagtttcttgccaccgccaactgtcaaatcagccctgttaaacattaaaagaaaacacttattttttgattttaagtttaaagccaaatacttagcatttatttcctgtctgttagagaaacctgatttatctgtaaaaacagctttaaagtcgattttcaggaaaagtcaggtcaggtcaatttcggaaaaattcggtttaaaccttaatgctcaaattgtttgtttgtctccaagtcaatggttaaactgttttttggaaatgctggaagttgtccctgaaaaatttcatccttcgtagttcaaagtcgggtggttgtcaagatgatttttttggtttggtgtcgtctttttttaagctgccgcataacggctggcaaattggcgatggagcggaaacgtaaaagaagttatggaaataagacttagaagcaaacttaagagtgtgttgatagtgcagtatcttaaaattttgtataataggaattgaagttaaattagatgctaaaaatttgtaattaagaaggagtgattacatgaacaaaaatataaaatattctcaaaactttttaacgagtgaaaaagtactcaaccaaataataaaacaattgaatttaaaagaaaccgataccgtttacgaaattggaacaggtaaagggcatttaacgacgaaactggctaaaataagtaaacaggtaacgtctattgaattagacagtcatctattcaacttatcgtcagaaaaattaaaactgaatactcgtgtcactttaattcaccaagatattctacagtttcaattccctaacaaacagaggtataaaattgttgggagtattccttaccatttaagcacacaaattattaaaaaagtggtttttgaaagccatgcgtctgacatctatctgattgttgaagaaggattctacaagcgtaccttggatattcaccgaacactagggttgctcttgcacactcaagtctcgattcagcaattgcttaagctgccagcggaatgctttcatcctaaaccaaaagtaaacagtgtcttaataaaacttacccgccataccacagatgttccagataaatattggaagctatatacgtactttgtttcaaaatgggtcaatcgagaatatcgtcaactgtttactaaaaatcagtttcatcaagcaatgaaacacgccaaagtaaacaatttaagtaccgttacttatgagcaagtattgtctatttttaatagttatctattatttaacgggaggaaataattctatgagtcgcttttgtaaatttggaaagttacacgttactaaagggaatgtagataaattattaggtatactactgacagcttcggggatcctctagagtcgacctgcag*TCTAACGGCGCATCGGCACGGGAGGATGTATTCCCATTCCGAGTCCATCGCGTCAAAGAAGTAGCCGTGCGCTACTTTCCGCACCTTACTCCGAATTCCGGTACACGTGCTCTGCGAAGAATCATCTATGGGGATCAAGATCTCCTCAATTCGATGAGAGAACATGGTTATGCTCTGGGACAGAGATCCTTGACACCGGCTATGCTCAACGTCCTTACAGCCTATCTTGGTTCGCCCGAAGATTTTTGTCCTTGACGGACAATAGCTGAAAGTCTGCTTATAGGGGGTATGTCGAAGTCGTGATTTGCGGCTTTCGGCATACCCCCTTTCAGTATATGCAAAGAGGATGTTTA |
| W83∆5140-Complement sequence |
| TAACGGTTCAAATTACGAATGCCCTTGGGGAAAACGATTCCTTTCTCTTCTCGAATCGTTGACTTTTCTGGTTATTCTGATTACTTAGATTCTTCCGATGCATAGTCGAAAAGATAAATATAGCCTTCACTGTTGATAACTCGGTGCGAAAAGTATCGGCAGATCGGTGCGAAATGT*ggtacccccgatagcttccgctattgcttttttgctcatcggtatttgcaacatcatagaaattgcatacctttgttcctcggttatatgtttgctcatctgcaacttttttttctttggacggacaattaaagcaaagatagcaaactttatccattcagagtgagagaaagggggacattgtctctctttcctctctgaaaaataaatgtttttattgcttattatccgcacccaaaaagttgcatttataagttgaactcaagaagtattcacctgtaagaagttactaatgacaaaaaagaaattgcccgttcgttttacgggtcagcactttactattgataaagtgctaataaaagatgcaataagacaagcaaatataagtaatcaggatacggttttagatattggggcaggcaaggggtttcttactgttcatttattaaaaatcgccaacaatgttgttgctattgaaaacgacacagctttggttgaacatttacgaaaattattttctgatgcccgaaatgttcaagttgtcggttgtgattttaggaattttgcagttccgaaatttcctttcaaagtggtgtcaaatattccttatggcattacttccgatattttcaaaatcctgatgtttgagagtcttggaaattttctgggaggttccattgtccttcaattagaacctacacaaaagttattttcgaggaagctttacaatccatataccgttttctatcatactttttttgatttgaaacttgtctatgaggtaggtcctgaaagtttcttgccaccgccaactgtcaaatcagccctgttaaacattaaaagaaaacacttattttttgattttaagtttaaagccaaatacttagcatttatttcctgtctgttagagaaacctgatttatctgtaaaaacagctttaaagtcgattttcaggaaaagtcaggtcaggtcaatttcggaaaaattcggtttaaaccttaatgctcaaattgtttgtttgtctccaagtcaatggttaaactgttttttggaaatgctggaagttgtccctgaaaaatttcatccttcgtagttcaaagtcgggtggttgtcaagatgatttttttggtttggtgtcgtctttttttaagctgccgcataacggctggcaaattggcgatggagcggaaacgtaaaagaagttatggaaataagacttagaagcaaacttaagagtgtgttgatagtgcagtatcttaaaattttgtataataggaattgaagttaaattagatgctaaaaatttgtaattaagaaggagtgattacatgaacaaaaatataaaatattctcaaaactttttaacgagtgaaaaagtactcaaccaaataataaaacaattgaatttaaaagaaaccgataccgtttacgaaattggaacaggtaaagggcatttaacgacgaaactggctaaaataagtaaacaggtaacgtctattgaattagacagtcatctattcaacttatcgtcagaaaaattaaaactgaatactcgtgtcactttaattcaccaagatattctacagtttcaattccctaacaaacagaggtataaaattgttgggagtattccttaccatttaagcacacaaattattaaaaaagtggtttttgaaagccatgcgtctgacatctatctgattgttgaagaaggattctacaagcgtaccttggatattcaccgaacactagggttgctcttgcacactcaagtctcgattcagcaattgcttaagctgccagcggaatgctttcatcctaaaccaaaagtaaacagtgtcttaataaaacttacccgccataccacagatgttccagataaatattggaagctatatacgtactttgtttcaaaatgggtcaatcgagaatatcgtcaactgtttactaaaaatcagtttcatcaagcaatgaaacacgccaaagtaaacaatttaagtaccgttacttatgagcaagtattgtctatttttaatagttatctattatttaacgggaggaaataattctatgagtcgcttttgtaaatttggaaagttacacgttactaaagggaaTG*ACCGCTCCATTATTTTGATGACATTGATTTTTGGAACATGAATAAAAGTTTATCTTTTTCGTTCATGCGGATATTATCAGAATAACCGCCTTTTGTTATTTGATACCCGCATGGCTTAACCATAAAAATGCCTAAGCCCTTAGTGTATGAACTTACTTCTGATGCATAGTCTTTACTTGTATTTAATGGAACTTTCCCTTTAATATGACACCACTCATTATTGCAACTGATGTCTTCAATCTCAGACATCATTTTTTGCAAATCTGTAATAGCTTTGGAACTTGCCGCTTGGGGTATCTGCAACTCAAAATAGAGCATCGGTTCGAGAATGTCCACACCTGACTGTTGCAAGGCCAGCCTGAAGACATAAGGGGTCAGCTGTCTGAAATCAGCAGGTGTACTTACCGGGCTATAATACTCGGCTTGAGTAAAAGTTACTTTCAGATCAGTCACTTCCCATCCATGTAACCCGGATTGGCAAGACATACGAATCCCTTCAAAAACGGCATTTTGAAAAGAATGGTTCAGATAACCATAGGAGATGTCACTTTCGATTTGCAACCCTGTCCCTAACGGTAAGGGTTCAAGAGTCAGCCCTATTGTGGCCCAATAAGGGTTGGGCGGCACTTCGATCTGAATAATCTTATTGACCTTTTTTACAGGTCGTTCTTTGTATATAGTCTTGATCTCATCAAAATGGACCTTTACGGAAAATCGTTCTTCCAGCAATGTCTGTATGATTTCCTTTTGGGTTAAACCATATAACGAGATTTCCAATTCATCACTATATGAGTTTATGGAAAAGGACAAAGACGGGTCTTCAATCCACAATGTATTCAGAGCGGATATCACCTTGCTTCTCTCTTCGGGCCTGTCTGGCCGGACGGAGGATTTGAGAGCGGGATGCTGATGCGATAATCCTTGAATCAAACAAGGTTCAGCACCTAAATAATTTCCGATTCGAAAATCATCCATATCCTCTACAATCGCGATATCATTGGCGCCCACTTCATCAACATTTATCTCTCTGCCCTGATTGATAGTTTTTAGATTTTTAATCTTGATGAATTTTTCCGAATCGTTGATTCTTACAACGTCTCGAAGTCTCAGACTTCCGTCAATTATTTTTAGAAAACTTCTTTTATGTCCTTTGGGGTCATGCTCTATCTTATAAAGATAAGATGAAAGTCTGTTTGAGACCGATGCCGGAGGAAGTATAAAAGAAGTGATGGCGTCCAACAACTCATTGATACCGATATTGAACATTGCTGATCCATGTAGCACCGGATAGACTTTGGCTTTTGCCACAAGAGCGATTATCGTATTCCAATAATCAGCCGGTGAAATTTCGCTATCCGCCAAATATCGTTCTAATATATTGTCGTCATGGTTGCATACAAATTCTTTGTATTCTTCCTTTATATATGTTTGGGAGCAAACCGGATAAACCGATCCATCGACAACATTTTGCATAAACAGGACATCTTGAGACAGATTTGCTTTTATATCCAGATACAAACGCTCCAAATTCACACCGGCTCGGTCAATCTTATTGATAAATATAATTGTCGGGATTTGCAGCTTCTGTAAAGTATTGAACAGCAACTTTGTCTGCGCTTGTATGCCTTCCTTTGCGGATAAGATGAGGACTGCTCCATCAAGCATTTTGAATGTCCGCTCCACTTCCGCAATAAAATCCATGTGTCCCGGAGTGTCAATGATATTGCATTTCACACCATTCCAGATAATAGATGTCGTAGAAGCCCGAACAGTAATTCCTCTACGTTTCTCTATATCCATAGAGTCCGTTATGGTGTCACCATTATCCACACAGCCGCACTTTTCCGTTGCTCCACTGGCAAACAGCAGATTCTCGGTTACGGAAGTTTTTCCTGCATCAATGTGAGCAAGAATTCCTAAATTTATAATATTCATTTGGATTAAGCAATAATATACTACAATAGATGCATTGTCGAAACGCACCTTTTAATACCTCCTCGTAGCATATGAGAACTACAGGATTCCTAACTCGTATTAATATGTATATTATTACTGCCGCATAACGATTACAAAATTACACAAAAAAATATATCTAACAAAAGTAGGAGGATTTTTTAACTTTTTACCATAGACATTTTATTAGGGAAACGTAGGTTCAACTGGTAAGTACAAATAAGTAGAAATGTTTGAACAACACTGTTTTAGGAAGTTGAAAAATCAAGTTTCTCTCTCGGTATAGCGTTTATTTTGGCCAATATCTTCTTTAGTTTGGCATTTGAGTATTTCCCATTCGTGTTCTATTTAGCTCCTTATTATGAGTATGTAGCAAGTTGCTTATCAAATTCAGCCTCTTTGAGGGTCAAATATGGTTTTGCAAATGGTGACTGACAAGACATAAACAAGGTCAGCAGGATTTTTTACATAAATGGACATGAATGTATATAACGGGGTTTGAGGGCCAA**CGGTTCAAATTACGAATGCCCTTGGGGAAAACGATTCCTTTCTCTTCTCGAATCGTTGACTTTTCTGGTTATTCTGATTACTTAGATTCTTCCGATGCATAGTCGAAAAGATAAATATAGCCTTCACTGTTGATAACTCGGTGCGAAAAGTATCGGCAGATCGGTGCGAAATGTATTCCTTTGTATTTGTCTGCCGGCAGGTGTAGCGATGCATTTTTCAGCCTGTTCGGTATTCATCATTATTCTATAAAAACACATACCAACATGAAAGTTAAATCTAACGGCGCATCGGCACGGGAGGATGTATTCCCATTCCGAGTCCATCGCGTCAAAGAAGTAGCCGTGCGCTACTTTCCGCACCTTACTCCGAATTCCGGTACACGTGCTCTGCGAAGAATCATCTATGGGGATCAAGATCTCCTCAATTCGATGAGAGAACATGGTTATGCTCTGGGACAGAGATCCTTGACACCGGCTATGCTCAACGTCCTTACAGCCTATCTTGGTTCGCCCGAAGATTTTTGTCCTTGACGGACAATAGCTGAAAGTCTGCTTATAGGGGGTATGTCGAAGTCGTGATTTGCGGCTTTCGGCATACCCCCTTTCAGTATATGCAAAGAGGATGTTTA** |

The start (green) and stop (red) codons are boxed. The following features are denoted in the sequence as described: The sequence containing the expressible *ErmF*(PG) / *ErmAM*(E.coli) cassette is italicized. The sequence containing the expressible *TetQ* gene is underlined and in opposite orientation to the *ErmF* gene. The completely restored intergenic region in the complement strain that codes for the PG_RS02100 sequence is in bold.
