# Supplementary material for: Deletion of a conserved transcript PG_RS02100 expressed during logarithmic growth in Porphyromonas gingivalis results in hyperpigmentation and increased tolerance to oxidative stress
Source: PLoS One. 2018 Nov 12;13(11):e0207295. doi: 10.1371/journal.pone.0207295 (PMC6231650; doi:10.1371/journal.pone.0207295)
Supplement: S1 Appendix — (DOCX) [file pone.0207295.s006.docx]

**S1 Appendix**

**Gingipain assay**

The gingipain assay used in this study was modified from the protocol described by Potempa and Nguyen (1),and is described in detail here. For a single 96-well plate, 750 μl of fresh substrate working solutions for Kgp activity (L-Lysine-p-nitroanilide dihydrobromide; Sigma L7002) and Rgp activity (BAPNA; Sigma B4875) were prepared. *P. gingivalis* strains were cultured in HR-media for 3 days anaerobically at 37˚C for maximal gingipain production, as it has been previously reported that gingipain protein maturation is maximal at stationary phase (2). 2 ml fractions from each culture were collected, OD_600nm_ determined (2.32 W83; 2.56 W83Δ514) and centrifuged at 5000 x g for 5 min at 4˚C. The spent media (cell-free supernatants) was reserved and filtered. Each cell pellet was suspended in 2 ml freshly prepared 1x assay buffer working solution and the OD_600nm_ determined (2.42 W83; 2.65 W83Δ514). 2-fold serial dilutions of spent media and cell suspensions were prepared in 1x assay buffer working solution. Cell suspensions were diluted to 100%, 50%, 25%, 12.5%, 6.25%, 3.125%. Spent media were diluted to 100%, 50%, 25%, 12.5%, 0% (negative control). Each well of a 96-well plate was prepared containing 90 μl 2x assay buffer working solution and 100 μl of sample and each plate contained two replicate wells. The wells were mixed by pipetting and the plate was incubated at 37˚C for 10 min to warm the samples and ensure reduction of cysteine residues. 10 μl of appropriate substrate solution was added to each sample well except the blanks and mixed thoroughly to produce a final concentration of 0.5 mM substrate. The absorbance at 410 nm was recorded using a temperature-regulated kinetic microplate reader and collected continuously over 2 to 30 min. To determine the initial rate of hydrolysis, the linear portion of the absorbance versus-time curve was calculated for each sample and adjusted by the rate of non-enzymatic substrate hydrolysis observed in the negative control wells. Since *P. gingivalis* does not significantly produce other Arg-specific or Lys-specific proteases (3), the rate of L-BAPNA and Ac-Lys-pNA hydrolysis is considered a direct measurement of gingipain activity. Moreover, the rate of L-BAPNA hydrolysis represents the sum of contributing forms of Rgp proteases expressed in *P. gingivalis*.

**References**

1. Potempa J, Nguyen KA. Purification and characterization of gingipains. Curr Protoc Protein Sci. 2007;Chapter 21:Unit 21 0.

2. Dou Y, Robles A, Roy F, Aruni AW, Sandberg L, Nothnagel E, et al. The roles of RgpB and Kgp in late onset gingipain activity in the vimA-defective mutant of Porphyromonas gingivalis W83. Molecular oral microbiology. 2015;30(5):347-60.

3. Shi Y, Ratnayake DB, Okamoto K, Abe N, Yamamoto K, Nakayama K. Genetic analyses of proteolysis, hemoglobin binding, and hemagglutination of Porphyromonas gingivalis. Construction of mutants with a combination of rgpA, rgpB, kgp, and hagA. J Biol Chem. 1999;274(25):17955-60.
