# Supplementary figures and images for: Deletion of a conserved transcript PG_RS02100 expressed during logarithmic growth in Porphyromonas gingivalis results in hyperpigmentation and increased tolerance to oxidative stress
Source: PLoS One. 2018 Nov 12;13(11):e0207295. doi: 10.1371/journal.pone.0207295 (PMC6231650; doi:10.1371/journal.pone.0207295)

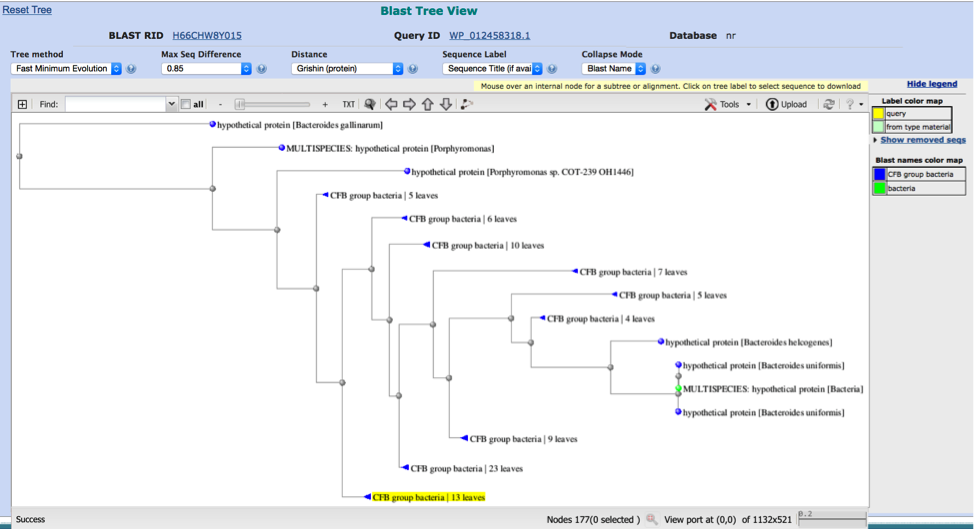

Supplement: S1 Fig — (TIFF) [file pone.0207295.s007.tiff]
